# Qualitative and Quantitative Analysis of Lignan Constituents in Caulis Trachelospermi by HPLC-QTOF-MS and HPLC-UV

**DOI:** 10.3390/molecules20058107

**Published:** 2015-05-05

**Authors:** Xiao-Ting Liu, Xu-Guang Wang, Yu Yang, Rui Xu, Fan-Hua Meng, Neng-Jiang Yu, Yi-Min Zhao

**Affiliations:** 1 Beijing Institute of Pharmacology and Toxicology, Beijing 100850, China; E-Mails: liuxiaoting_2013@163.com (X.-T.L.); xuguang_wang521@sina.com (X.-G.W.); tommase@sina.com (Y.Y.); beijingxurui@sina.com (R.X.); mengfh331@126.com (F.-H.M.); 2 School of Traditional Chinese Materia Medica, Shenyang Pharmaceutical University, Shenyang 110016, China

**Keywords:** Caulis Trachelospermi, HPLC-QTOF-MS, HPLC-UV, dibenzylbutyrolatone lignans

## Abstract

A high-performance liquid chromatography coupled with quadrupole tandem time-of-flight mass (HPLC-QTOF-MS) and ultraviolet spectrometry (HPLC-UV) was established for simultaneous qualitative and quantitative analysis of the major chemical constituents in Caulis Trachelospermi, respectively. The analysis was performed on an Agilent Zorbax Eclipse Plus C18 column (4.6 mm × 150 mm, 5 μm) using a binary gradient system of water and methanol, with ultraviolet absorption at 230 nm. Based on high-resolution ESI-MS/MS fragmentation behaviors of the reference standards, the characteristic cleavage patterns of lignano-9, 9'-lactones and lignano-8'-hydroxy-9, 9'-lactones were obtained. The results demonstrated that the characteristic fragmentation patterns are valuable for identifying and differentiating lignano-9,9'-lactones and lignano-8'-hydroxy-9,9'-lactones. As such, a total of 25 compounds in Caulis Trachelospermi were unambiguously or tentatively identified via comparisons with reference standards or literature. In addition, 14 dibenzylbutyrolatone lignans were simultaneously quantified in Caulis Trachelospermi by HPLC-UV method. The method is suitable for the qualitative and quantitative analyses of dibenzylbutyrolatone lignans in Caulis Trachelospermi.

## 1. Introduction

Caulis Trachelospermi, the stems and leaves of *Trachelospermum jasminoides* (Lindl.) Lem, is mainly distributed in Henan, Anhui, Hubei, Shandong and Guangxi provinces in China. It has been used in traditional Chinese medicine for the treatment of rheumatic arthralgia, aching of the loins and knees, traumatic injuries [1], and its medicinal properties such as anticancer and anti-inflammation have been reported [2,3]. Chemical investigations indicated that it mainly contains lignans, flavonoids and triterpenoids [4,5,6]. In our previous study, the extract of Caulis Trachelospermi and its main dibenzylbutyrolactone lignan constituents exhibited marked anti-inflammatory activity in animal model [7], moderate inhibiting activity on NF-κB signaling pathway induced by TNF-α [8] as well as strong inhibiting activity on JAK/STAT pathway [9]. As mentioned above, the major bioactive constituents of Caulis Trachelospermi are disclosed to be dibenzylbutyrolactone lignans.

Up to now, high-performance liquid chromatography (HPLC) [10,11,12,13,14,15,16] and ultraviolet spectrophotometry (UV) [17,18] have been developed and focused on the quantitative analysis of total flavonoid, total lignans and a few active compounds such as trachelogenin and tracheloside in Caulis Trachelospermi. However, the fingerprint analysis in our previous research [19] has led to the discovery of more than 15 characteristic peaks, the content of which is still unequivocal. To the best of our knowledge, there have been no reports for the simultaneous determination of 14 main dibenzylbutyrolactone lignans by HPLC so far. Therefore, it is necessary to develop a sensitive and selective method to quantify the dibenzylbutyrolactone lignan constituents in Caulis Trachelospermi.

Currently, HPLC coupled with quadrupole time-of-flight mass spectrometry (QTOF-MS) is used in composition analysis and quantification of a wide variety of natural product compounds [20,21,22]. On the other hand, QTOF allows the generation of mass information with greater accuracy and precision, and it provides both elemental compositions and fragmentation patterns in a highly sensitive and convenient way [23]. However, no attempts have been made to identify the constituents in Caulis Trachelospermi based on accurate mass measurements using HPLC-QTOF-MS.

**Figure 1:**
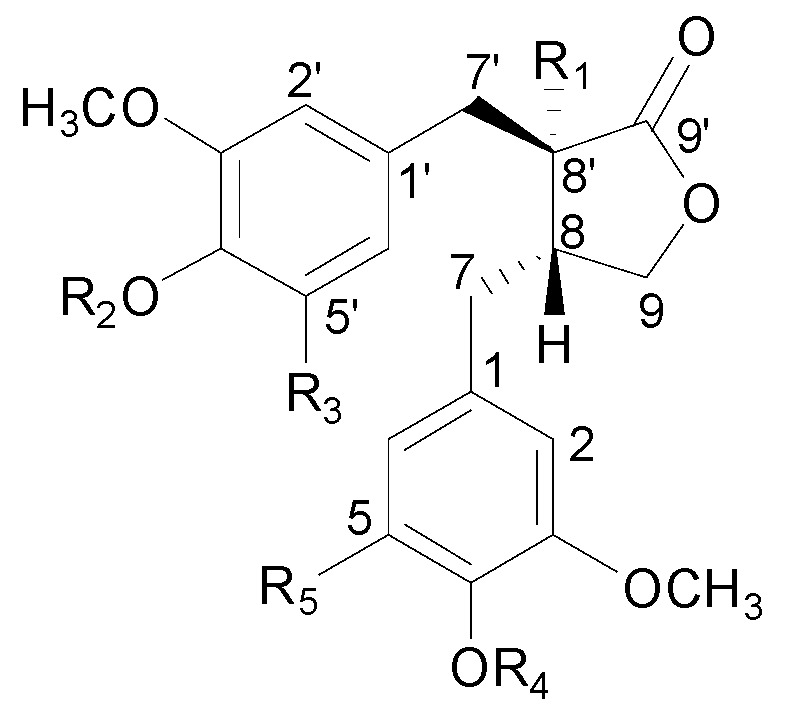

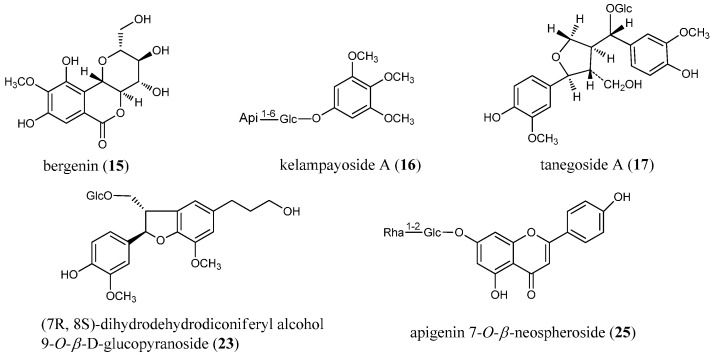
Chemical structures of compounds identified in Caulis Trachelospermi. Glc: glucose, Api: apiose, Rha: rhamnose.

In the present study, a combinative method using HPLC-QTOF-MS and HPLC-UV was first established for the identification and simultaneous quantification of the major constituents whose chemical structures are presented in Figure 1 in 14 batch samples of Caulis Trachelospermi, which could provide an alternative, feasible approach for the quality assessment of Caulis Trachelospermi.

**Table:** 

No.	Compound Name	R_1_	R_2_	R_3_	R_4_	R_5_
1	nortrachelogenin 5'-*C*-*β*-d-glucoside	OH	H	Glc	H	H
2	nortracheloside	OH	Glc	H	H	H
3	nortrachelogenin 8'-*O*-*β*-d-glucoside	O-Glc	H	H	H	H
4	matairesinol 4'-*O*-*β*-gentiobioside	H	Glc ^6-1^Glc	H	H	H
5	trachelogenin 4'-*O*-*β*-gentiobioside	OH	Glc ^6-1^Glc	H	CH_3_	H
6	matairesinoside	H	Glc	H	H	H
7	tracheloside	OH	Glc	H	CH_3_	H
8	arctigenin 4'-*O*-*β*-gentiobioside	H	Glc ^6-1^Glc	H	CH_3_	H
9	nortrachelogenin	OH	H	H	H	H
10	arctiin	H	Glc	H	CH_3_	H
11	matairesinol	H	H	H	H	H
12	trachelogenin	OH	H	H	CH_3_	H
13	5-methoxytrachelogenin	OH	H	H	CH_3_	OCH_3_
14	arctigenin	H	H	H	CH_3_	H
18	nortrachelogenin 4,4'-di-*O*-*β*-d-glucoside	OH	Glc	H	Glc	H
19	matairesinol 4,4'-di-*O*-*β*-d-glucoside	H	Glc	H	Glc	H
20	nortrachelogenin 4'-*O*-*β*-gentiobioside	OH	Glc ^6-1^Glc	H	H	H
21	nortrachelogenin 4-*O*-*β*-d-glucoside	OH	H	H	Glc	H
22	4-demethyltraxillaside	H	Glc	H	H	OCH_3_
24	traxillageside	H	Glc	H	OCH_3_	OCH_3_

## 2. Results and Discussion

### 2.1. Quantitative Analysis of Dibenzylbutyrolatone Lignans 

#### 2.1.1. Validation of the Developed Method

The quantification method was validated in terms of linearity, LODs and LOQs, precision, repeatability, stability, and accuracy. The results are listed in Table 1 and Table 2. All calibration curves showed good linearity (r^2^ > 0.9997) within the test ranges, and the LODs and LOQs were 1.24–9.00 and 3.71–31.71 ng, respectively. The intra and inter-day precision of the standard solutions were found in the range of 0.17%–0.75% and 0.15%–2.87%, respectively. Both for repeatability and stability test, the RSD were less than 2.94% and 2.78%, respectively. Recovery was between 96.68% and 103.63% with RSD values below 1.87%. These validation results indicated that the present method was sensitive, precise, repeatable, stable and accurate for the quantitative analysis of 14 dibenzylbutyrolatone lignans in Caulis Trachelospermi.

**Table 1:** Linear regression, LOD and LOQ, intra-day and inter-day precisions of the 14 dibenzylbutyrolatone lignans.

Compound	Regression Equation ^b^	R^2^	Range (ng)	LOD (ng)	LOQ (ng)	Intra-day (RSD%, *n* = 6)	Inter-day (RSD%, *n* = 6)
1 ^a^	*y* = 0.9845*x* − 3.0467	1.0000	36.18–1266.30	4.27	8.54	0.33	1.70
2	*y* = 1.2843*x* − 12.4895	1.0000	91.80–3213.00	9.00	18.01	0.44	0.15
3	*y* = 1.0255*x*− 11.8232	0.9999	39.03–1366.05	8.37	18.30	0.42	0.41
4	*y* = 0.8371*x* − 4.3034	1.0000	46.29–1620.15	4.99	14.97	0.33	0.26
5	*y* = 1.1938*x* − 4.7773	1.0000	86.31–3020.85	1.39	6.95	0.17	0.47
6	*y* = 0.9789*x* − 15.6369	1.0000	91.60–3206.00	6.25	27.49	0.44	0.85
7	*y* = 1.8986*x* + 12.2728	1.0000	280.56–9819.60	2.94	12.73	0.28	0.42
8	*y* = 1.0888*x* − 3.4518	1.0000	107.38–3758.30	7.93	31.74	0.24	0.46
9	*y* = 1.7141*x* − 29.0498	1.0000	61.05–2136.75	8.06	24.93	0.22	0.41
10	*y* = 1.2220*x* − 1.4093	1.0000	86.40–3024.00	7.72	24.95	0.31	0.85
11	*y* = 1.0257*x* − 24.0442	0.9997	49.50–1732.50	1.24	3.71	0.19	0.48
12	*y* = 2.1915*x* − 41.1740	1.0000	125.20–4382.00	3.13	10.42	0.22	0.43
13	*y* = 1.5909*x* − 0.0134	1.0000	12.35–432.25	4.06	13.95	0.17	2.87
14	*y* = 1.7761*x* − 4.4637	0.9999	22.76–796.43	3.35	11.18	0.75	2.22

^a^ The compounds are the same as in Figure 1; ^b^*y* is the peak area, *x* is the concentration (ng) of compound.

**Table 2:** Repeatability, stability and recovery of 14 dibenzylbutyrolatone lignans in Caulis Trachelospermi.

Compound	RSD (%, *n* = 6)		Recovery (%, *n* = 6)				
	Repeatability	Stability	Original (μg)	Spiked (μg)	Observed (μg)	Mean	RSD (%)
1	0.71	1.51	163.01	164.02	329.03	101.22	1.02
2	0.46	0.31	343.80	362.30	706.23	100.04	0.24
3	2.94	0.85	127.43	143.63	273.94	102.01	1.87
4	0.89	2.08	170.29	175.29	344.42	99.33	0.45
5	0.41	1.23	375.40	335.38	718.15	102.20	0.75
6	2.07	2.78	483.13	474.85	946.76	97.64	1.09
7	0.52	0.28	1325.26	1242.48	2542.17	97.94	0.34
8	0.70	1.19	224.28	257.71	478.29	98.56	0.38
9	0.90	1.03	198.16	234.43	429.08	98.51	0.17
10	0.76	2.21	421.27	421.63	858.20	103.63	0.39
11	1.47	0.80	292.02	277.20	575.80	102.37	0.30
12	1.12	0.26	439.82	420.67	859.56	99.78	0.27
13	2.70	1.14	15.88	20.75	36.93	101.43	0.71
14	1.98	1.00	89.00	87.38	174.48	96.68	0.48

#### 2.1.2. Sample Analysis

**Figure 2:**
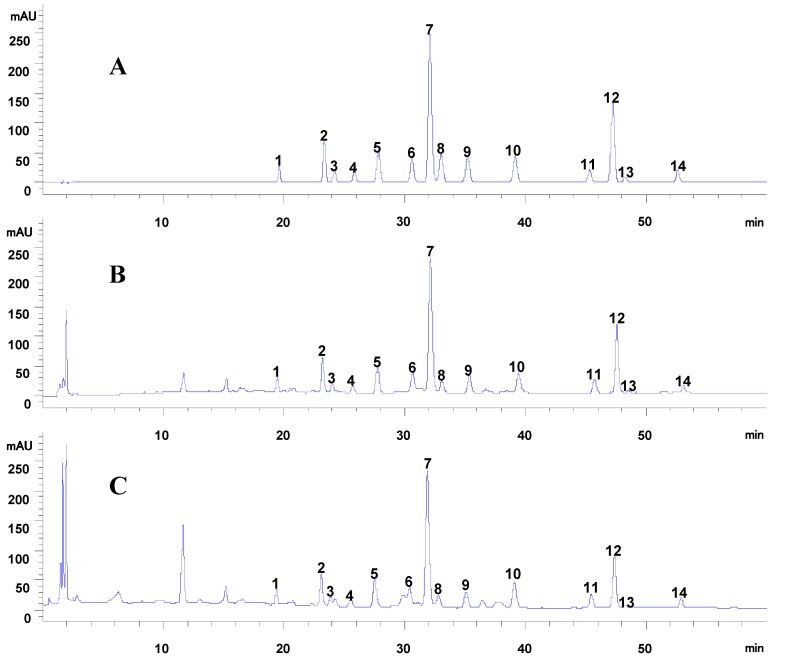
(**A**) HPLC-UV chromatogram of mixed standards. (**B**) HPLC-UV chromatogram of the sample extracted from Caulis Trachelospermi. (**C**) HPLC-UV chromatogram of Caulis Trachelospermi. The number of peaks marked in Figure 2 is corresponding to Figure 1.

The developed method was successfully applied to the simultaneous determination of 14 dibenzylbutyrolatone lignans in 14 batches of Caulis Trachelospermi. Representative chromatograms are shown in Figure 2C. Quantification of each compound in the samples was calculated with the external standard using the calibration curves. Information regarding the content is summarized in Table 3. According to Table 3, all the 14 compounds were detected from the 14 batches of Caulis Trachelospermi samples. Their contents varied dramatically with RSD (%) ranging from 30.54%–61.54%, but the variation of total content of all compounds was not that large. The average total content of these 14 compounds was 28.531 mg/g. The results also showed that in all samples, tracheloside was the maximal constituent, with a mean content of 7.237 mg/g. Besides, as shown in Table 3, most compounds in Y2-4 revealed relatively lower contents than others, and the contents of Y2-12 differed greatly from other batches. This was probably caused by the poor native quality of the analyzed samples. In general, the full scale multiple compounds quantification method developed in this paper has not been provided so far which will shed some new light on the quality control of Caulis Trachelospermi.

**Table 3:** Content (*n* = 3, mg/g) of 14 dibenzylbutyrolatone lignans in the tested samples.

Sample	1	2	3	4	5	6	7	8	9	10	11	12	13	14	Sum
Y2-1	0.785	1.706	0.667	0.758	1.949	2.121	6.602	1.130	0.932	2.184	1.388	2.041	0.092	0.405	22.761
Y2–2	1.355	4.712	1.397	1.639	2.643	5.294	11.301	2.136	0.966	2.678	1.440	1.833	0.116	0.423	37.934
Y2–3	1.068	3.394	2.013	1.757	1.533	5.627	7.218	1.808	0.313	3.166	0.584	0.917	0.048	0.348	29.795
Y2–4	0.706	1.032	0.735	0.306	0.333	1.172	2.777	0.314	1.043	1.155	1.171	2.222	0.147	0.303	13.415
Y2–5	0.989	2.491	1.113	0.540	1.327	2.408	8.054	1.040	1.517	2.579	1.498	3.505	0.159	0.484	27.703
Y2–6	1.000	3.984	0.622	0.563	1.378	2.113	8.742	0.814	1.932	0.570	1.409	3.422	0.303	0.259	27.112
Y2–7	1.402	3.601	1.427	1.585	2.748	4.234	10.339	1.769	1.116	2.047	1.554	2.255	0.161	0.413	34.650
Y2–8	1.080	4.098	1.108	1.742	2.698	5.124	10.344	2.377	0.955	2.256	1.383	2.003	0.131	0.453	35.751
Y2–9	0.676	1.029	0.862	0.616	1.314	1.829	4.505	0.805	1.381	1.839	2.251	4.168	0.210	0.664	22.148
Y2–10	0.927	4.273	1.931	2.174	1.625	6.832	8.613	2.021	0.471	3.789	0.865	1.314	0.056	0.573	35.464
Y2–11	0.805	3.544	1.234	1.105	1.414	3.796	7.718	1.280	1.262	1.878	0.847	1.534	0.099	0.442	26.958
Y2–12	0.397	0.984	0.779	0.608	0.606	1.364	3.054	0.677	1.267	0.701	2.360	3.906	0.247	1.644	18.594
Y2–13	0.827	2.164	1.239	1.070	1.498	2.774	7.275	1.045	2.546	1.484	3.102	5.335	0.348	0.900	31.607
Y2–14	0.616	0.998	0.895	0.660	1.398	1.744	4.775	0.943	1.341	1.736	2.279	4.352	0.206	0.810	22.752
Average	0.902	2.715	1.145	1.080	1.605	3.317	7.237	1.297	1.217	2.004	1.581	2.772	0.166	0.580	28.531
RSD (%)	30.54	50.94	38.17	54.69	44.48	54.86	36.79	47.74	45.80	44.37	43.69	47.92	53.41	61.54	26.20

### 2.2. Qualitative Analysis of Caulis Trachelospermi

#### 2.2.1. Fragmentation Characteristics of Dibenzylbutyrolatone Lignans

**Figure 3:**
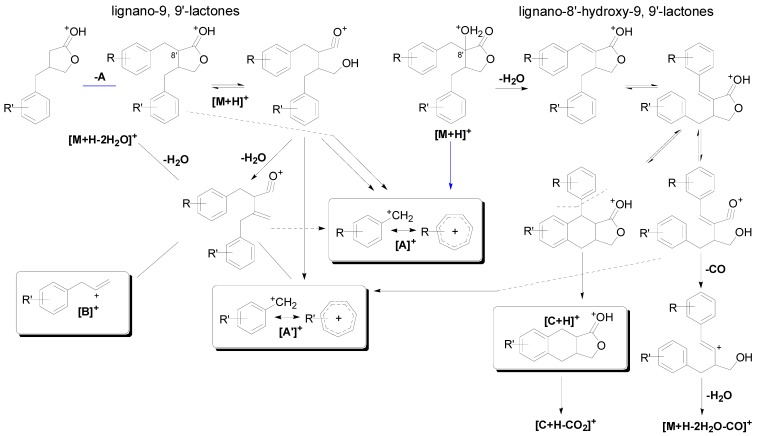
MS/MS fragmentation pathways of lignano-9, 9'-lactones and lignano-8'-hydroxy-9, 9'-lactones.

The mass spectra of reference compounds indicated that the accurate molecule weight of the quasi-molecule ions was highly consistent with that of the calculated ones (see Table S1). Thus, the molecule formula of the compounds in the sample can be uniquely deduced with the accurate molecule weight. In the MS spectra, all dibenzylbutyrolatone lignan standards showed strong [M+Na]^+^ signals in the positive ion mode. The selected precursor ions were dissociated using MS/MS to generate a series of abundant fragment ions. According to the MS data, the fragmentation patterns of lignano-9, 9'-lactones and lignano-8'-hydroxy-9, 9'-lactones exhibited diagnostic distinction between each other. The fragmentation pathways are summarized in Figure 3 and characteristic fragment ions of reference standards in MS/MS spectra are shown in Table 4 and Table 5.

The fragmentation pathways (Figure 3) of lignano-9, 9'-lactones such as matairesinol were in agreement with Schmidt’s research that the [A]^+^ ion, the analogous product ion [A']^+^ and the ion [B]^+^ is observed with significant abundance [24]. Furthermore, the high-resolution MS experiment of our research provides sufficient confirmation for Schmidt’s conclusions. The formation of two characteristic fragments ([A]^+^ and [B]^+^) provides information allowing the distinction between isomers with exchanged substitution of the two benzyl moieties [25]. Notably, the fragment ion at *m*/*z* 223.0968 (corresponding to [M+H−A]^+^ of matairesinol) which was first observed in our research can also determine the substitution of the two benzyl moietites of lignano-9,9'-lactones. Thus, the ion [M+H−A]^+^ can be interpreted as a complementary of [B]^+^ for the characteristic product ions.

The MS/MS spectrum of trachelogenin which is representative of lignano-8'-hydroxy-9,9'-lactones generated an abundant fragment at *m/z* 371.1496 [M+H−H_2_O]^+^ to yield a lign-7-eno-9,9'-lactones intermediate. The subsequent fragmentation pathways are identical with that of lign-7-eno-9,9'-lactones proposed by Schmidt *et al.* [25] and very abundant ions [C+H]^+^ and [A']^+^ were observed. However, fragmentary ion at *m*/*z* 137.0613 (termed [A]^+^ in Figure 3) which has not been reported by Schmidt *et al.* in lign-7-eno-9,9'-lactones can also be detected. Consequently, the fragment described above is of diagnostic value in the straightforward assignment of aromatic substitution in lignano-8'-hydroxy-9,9'-lactones.

In the MS/MS spectra of lignano-9,9'-lactone and lignano-8'-hydroxy-9,9'-lactone *O*-glycosides, ion [M+H−162]^+^ or [M+H−162−162]^+^ was obtained after eliminating the glucose residue (−162 Da) and subsequent MS behavior is in line with the typical fragmentation pathways of the aglycone moieties except matairesinol 4'-*O*-*β*-gentiobioside (**4**). On the other hand, nortrachelogenin 5'-*C*-*β*-d-glucoside (**1**) showed the characteristic fragment ions at 441.1555 [M+H−2H_2_O−60]^+^ and 423.1459 [M+H−3H_2_O−60]^+^ which was result from a cross-link cleavage of the C-glycoside moiety. Unfortunately, the diagnostic ion cannot be detected in 5-methoxytrachelogenin (**13**) and arctigenin (**14**). It was likely due to the inadequate MS/MS conditions or some unknown factors which requires further investigation.

Overall, the characteristic [B]^+^ and [C+H]^+^ ions in combination with the [A]^+^ and [A']^+^ ions allows the unambiguous identification and distinction between lignano-9,9'-lactones and lignano-8'-hydroxy-9,9'-lactones.

**Table 4:** Key MS/MS fragmentation data of reference lignano-9,9'-lactones compounds.

Peak No.	[A]^+^ (Measured)	[A]^+^ (Calculated)	Error (ppm)	[A']^+^ (Measured)	[A']^+^ (Calculated)	Error (ppm)	[B]^+^ (Measured)	[B]^+^ (Calculated)	Error (ppm)	[M+H−A]^+^ (Measured)	[M+H−A]^+^ (Calculated)	Error (ppm)
4	137.0603	137.0603	0	137.0603	137.0603	0						
6	137.0599	137.0603	−2.92	137.0599	137.0603	−2.92	163.0755	163.0754	0.61	223.0973	223.0965	3.55
8	137.0601	137.0603	−1.46							237.1117	237.1121	−1.69
10	137.0604	137.0603	0.73	151.0759	151.0754	3.31	177.0923	177.0910	7.34	237.1127	237.1121	2.53
11	137.0607	137.0603	2.92	137.0607	137.0603	2.92	163.0762	163.0762	4.91	223.0968	223.0965	1.35
14	137.0599	137.0603	−2.92	151.0753	151.0754	−0.66						

**Table 5:** Key MS/MS fragmentation data of reference lignano-8'-hydroxy-9,9'-lactones compounds.

Peak No.	[A]^+^ (Measured)	[A]^+^ (Calculated)	Error (ppm)	[A']^+^ (Measured)	[A']^+^ (Calculated)	Error (ppm)	[C+H]^+^ (Measured)	[C+H]^+^ (Calculated)	Error (ppm)
1				137.0607	137.0603	2.92			
2	137.0610	137.0603	5.12	137.0610	137.0603	5.12	233.0816	233.0808	3.43
3	137.0602	137.0603	−0.73	137.0602	137.0603	−0.73	233.0812	233.0808	1.72
5	137.0619	137.0603	11.67	151.0775	151.0754	13.90	247.0982	247.0965	6.88
7	137.0605	137.0603	1.46	151.0763	151.0754	5.96	247.0973	247.0965	3.24
9	137.0614	137.0603	8.03	137.0614	137.0603	8.03	233.0820	233.0808	5.15
12	137.0613	137.0603	7.30	151.0770	151.0754	10.59	247.0977	247.0965	4.86
13	137.0613	137.0603	7.30	181.0861	181.0859	1.10			

#### 2.2.2. Identification of Constituents in the Sample Extracted from Caulis Trachelospermi

**Figure 4:**
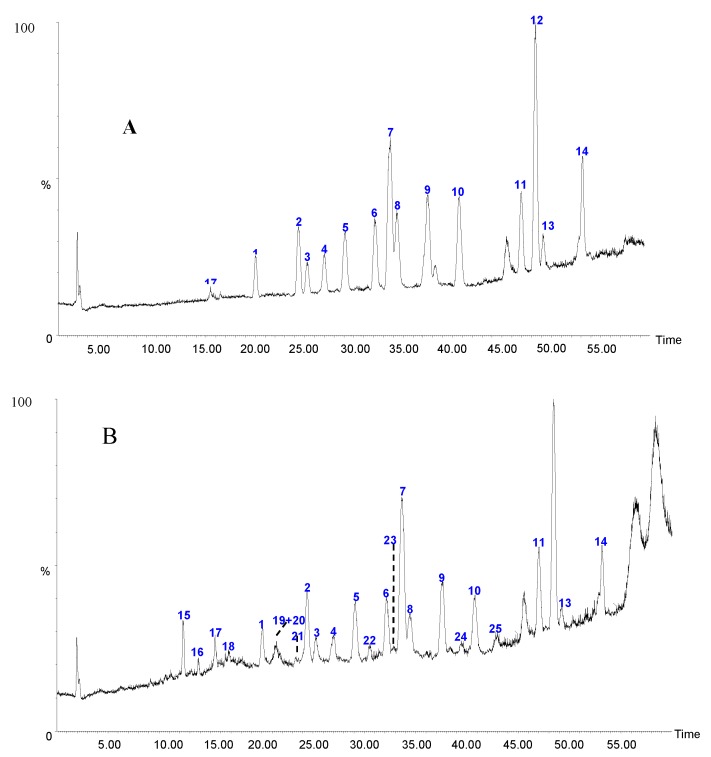
(**A**) Total ion current (TIC) of mixed standards. (**B**) Total ion current (TIC) of the sample extracted from Caulis Trachelospermi. The number of peaks marked in Figure 4 is corresponding to Figure 1.

Before qualitative analysis of the constituents in Caulis Trachelospermi by HPLC-QTOF-MS, purification from Caulis Trachelospermi was performed by HP-20 macroporous resin column chromatography to obtain an extract for analysis in order to reduce the matrix interference. As shown in the HPLC-UV chromatogram (Figure 2B), the sample after purification successfully remains the major constituents in Caulis Trachelospermi. Its total ion chromatogram (TIC) from HPLC-QTOF-MS analysis is shown in Figure 4. Under the present chromatographic and MS conditions, in total 25 compounds were detected (as shown in Table 6 and Figure 4). Among them, 15 compounds were unambiguously identified by comparing with the retention time and MS data of reference standards.

Compound **21** showed an accurate mass of [M+Na]^+^ ion at *m*/*z* 559.1808 corresponding to the molecular formula C_26_H_32_O_12_. In the MS/MS spectrum, signals for ion at *m*/*z* 397.1328 [M+Na−162]^+^ via the loss of glucose and the characteristic ion at 159.0415 [A−H+Na]^+^ implied that it was nortrachelogenin glucoside. According to the different LC retention behaviors from the known isomers nortracheloside (**2**) and nortrachelogenin 8'-*O-β*-d-glucoside (**3**), it was assigned as nortrachelogenin 4-*O-β*-d-glucoside. Similarly, compound **18**, **22** and **24** was deduced to be nortrachelogenin 4, 4'-di-*O-β-*d-glucoside, 4-demethyltraxillaside and traxillageside, respectively. Their structures were confirmed by NMR techniques after isolaton and purification in our previous research [9,26]. 

**Table 6:** Key MS/MS fragmentation data of other constituents in the sample extraced from Caulis Trachelospermi.

Peak No.	t_R_ (min)	Precursorion (*m/z*)	Error (ppm)	Formula	Fragments (*m/z*)	Elem. comp.	Pathways	Identity
15	12.349	329.0865 [M+H]^+^	−2.43	C_14_H_16_O_9_	293.0663 275.0549 263.0555 247.0610 233.0442	C_14_H_13_O_7_^+^ C_14_H_11_O_6_^+^ C_13_H_11_O_6_^+^ C_13_H_11_O_5_^+^ C_12_H_9_O_5_^+^	[M+H−2H_2_O]^+^ [M+H−3H_2_O]^+^ [M+H−2H_2_O−HCOH]^+^ [M+H−3H_2_O−CO]^+^ [M+H−2H_2_O−CH_3_COOH]^+^	bergenin
16	13.795	501.1551 [M+Na]^+^	−5.79	C_20_H_30_O_13_	411.1230 369.1147	C_17_H_24_O_10_Na^+^ C_15_H_22_O_9_Na^+^	[M+Na−3HCOH]^+^ [M+Na−Api]^+^	kelampayoside A
17	15.450	561.1963 [M+Na]^+^	2.63	C_26_H_34_O_12_	381.1310	C_20_H_22_O_6_Na^+^	[M+Na−Glc−H_2_O]^+^	tanegoside A
18	16.775	721.2315 [M+Na]^+^	−0.69	C_32_H_42_O_17_	559.1771 397.1249 159.0419	C_26_H_32_O_12_Na^+^ C_20_H_22_O_7_Na^+^ C_8_H_8_O_2_Na^+^	[M+Na−Glc]^+^ [M+Na−2Glc]^+^ [A−H+Na]^+^/[A'−H+Na]^+^	nortrachelogenin 4,4'-di- *O-β-*d-glucoside
19	21.416	705.2371 [M+Na]^+^	0	C_32_H_42_O_16_	543.1870 381.1335 159.0435	C_26_H_32_O_11_Na^+^ C_20_H_22_O_6_Na^+^ C_8_H_8_O_2_Na^+^	[M+Na−Glc]^+^ [M+Na−2Glc]^+^ [A−H+Na]^+^/[A'−H+Na]^+^	matairesinol 4,4'-di- *O-β-*d-glucoside
20	21.416	721.2309 [M+Na]^+^	−1.53	C_32_H_42_O_17_	559.1743 397.1295 159.0421	C_26_H_32_O_12_Na^+^ C_20_H_22_O_7_Na^+^ C_8_H_8_O_2_Na^+^	[M+Na−Glc]^+^ [M+Na−2Glc]^+^ [A−H+Na]^+^/[A'−H+Na]^+^	nortrachelogenin 4'- *O*-*β*-gentiobioside
21	22.976	559.1792 [M+Na]^+^	0.18	C_26_H_32_O_12_	397.1328 159.0415	C_20_H_22_O_7_Na^+^ C_8_H_8_O_2_Na^+^	[M+Na−Glc]^+^ [A−H+Na]^+^/[A'−H+Na]^+^	nortrachelogenin 4- *O*-*β-*d-glucoside
22	30.531	573.1968 [M+Na]^+^	3.49	C_27_H_34_O_12_	411.1426	C_21_H_24_O_7_Na^+^	[M+Na−Glc]^+^	4-demethyltraxillaside
23	32.928	545.1992 [M+Na]^+^	−1.28	C_26_H_34_O_11_	383.1596 159.0366	C_20_H_24_O_6_Na^+^ C_8_H_8_O_2_Na^+^	[M+Na−Glc]^+^ [A−H+Na]^+^	dihydrodehydrodiconiferyl alcohol-9- *O*-*β*-d-glucoside
24	39.434	587.2125 [M+Na]^+^	3.58	C_28_H_36_O_12_	425.1583 159.0412	C_22_H_26_O_7_Na^+^ C_8_H_8_O_2_Na^+^	[M+Na−Glc] ^+^ [A−H+Na]^+^	traxillageside
25	42.339	579.1719 [M+H]^+^	0.86	C_27_H_30_O_14_	433.1125 271.0605	C_21_H_21_O_10_^+^ C_15_H_11_O_5_^+^	[M+H−Rha]^+^ [M+H−Rha−Glc]^+^	apigenin 7- *O*-*β*-neospheroside

Compound **20** yielded product ions at 559.1743 [M+Na−162]^+^ and 397.1295 [M+Na−162−162]^+^ which correspond to the aglycone form after loss of hexosyl moiety, together with the fragment ion at 159.0421 [A−H+Na]^+^, sugguesting the aglycone moiety is nortrachelogenin. According to the biogenetic regularity that the sugar residue is mostly glucosyl or gentiobiosyl residue and sugar residue is preferentially connected to C-4', compound **20** was deduced as nortrachelogenin 4, 4'-di-*O-β-*d-glucoside or nortrachelogenin 4'-*O*-*β*-gentiobioside. Since its LC retention time is different from that of the known isomer nortrachelogenin 4, 4'-di-*O-β-*d-glucoside (**18**), its structure was assigned as nortrachelogenin 4'-*O*-*β*-gentiobioside. Similarly, Compound **19** was deduced as matairesinol 4, 4'-di-*O*-*β*-d-glucoside according to its MS data and different LC retention time from that of its known isomer matairesinol 4'-*O*-*β*-gentiobioside (**4**).

Other compounds (**15**, **16**, **23** and **25**) were tentatively identified by comparing MS data and LC retention behavior with the literature published by our group [9,26,27].

However, the dibenzylbutyrolatone lignans with low content in sample did not show key information such as the characteristic ions [B]^+^ and [C+H]^+^ having critical importance in structural elucidation of dibenzylbutyrolatone lignans. This was probably due to the trace content of these compounds which were easily interfered with by matrix.

## 3. Experimental Section 

### 3.1. Chemicals, Reagents and Materials

Reference standards (Figure 1), nortrachelogenin 5'-*C*-*β*-d-glucoside (**1**), nortracheloside (**2**), nortrachelogenin 8'-*O*-*β*-d-glucoside (**3**), matairesinol 4'-*O*-*β*-gentiobioside (**4**), trachelogenin 4'-*O*-*β*-gentiobioside (**5**), matairesinoside (**6**), tracheloside (**7**), arctigenin 4'-*O*-*β*-gentiobioside (**8**), nortrachelogenin (**9**), arctiin (**10**), matairesinol (**11**), trachelogenin (**12**), 5-methoxytrachelogenin (**13**), arctigenin (**14**) and tanegoside A (**17**) were isolated from Caulis Trachelospermi and identified in our previous research [8,26,27,28]. The purities of the standards were determined to be above 95% by normalization of the peak areas detected by HPLC analyses.

HPLC grade methanol was obtained from Fisher Scientific (Fair Lawn, NJ, USA). Purified water was purchased from Wahaha Ltd. (Hangzhou, China). Other reagents, purchased from Sinopharm Chemical Reagent Co. Ltd. (Beijing, China) were of analytical grade. HP-20 macroporous resin was purchased from Mitsubishi Chemical Co. (Tokyo, Japan). 14 batches of Caulis Trachelospermi were acquired from different pharmaceutical companies in China (Table 7). All of the samples were authenticated by senior engineer Qi-yun Ma, Beijing Institute of Pharmacology and Toxicology, Beijing, China. Voucher specimens were deposited in the Department of Natural Products Chemistry, Beijing Institute of Pharmacology and Toxicology, Beijing, China. Batch no. 080130 (purchased from Beijing Qijing Chinese Herbs Factory, Beijing, China in 2008) was selected as the sample for method validation.

**Table 7:** Sample information for 14 batches Caulis Trachelospermi.

Sample no.	Batch no.	Source
Y2–1	080130	Zhejiang (QJ ^a^)
Y2–2	0511011	Zhejiang (CG)
Y2–3	060530	Anhui (SF)
Y2–4	061329	Anhui (SL)
Y2–5	05090101	Shandong (PSL)
Y2–6	05081205	Zhejiang (QJ)
Y2–7	20030928	Yunnan (BTS)
Y2–8	20060415	Yunnan (BTS)
Y2–9	5050028	Guangxi (TRT)
Y2–10	1040804	Henan (TRT)
Y2–11	060626	Jiangsu (LRT)
Y2–12	070301	Zhejiang (XD)
Y2–13	F060290	Sichuan (DRT)
Y2–14	20070102	Jiangsu (QX)

^a^ Abbreviated for different pharmaceutical companies.

### 3.2. Standard Solutions Preparation

Individual stock solutions of reference standards were prepared by accurately weighing into a 10 mL volumetric flask and dissolving the reference compounds in methanol. A mixed solution used for quantitative analysis was prepared by placing a certain amount of each stock solution in a 10 mL volumetric flask and diluted to volume with 50% methanol aqueous solution at the concentration of 36.18 μg∙mL^−1^ nortrachelogenin 5'-*C*-*β*-d-glucoside (**1**), 91.80 μg∙mL^−1^ nortracheloside (**2**), 39.03 μg∙mL^−1^ nortrachelogenin 8'-*O*-*β*-d-glucoside (**3**), 46.29 μg∙mL^−1^ matairesinol 4'-*O*-*β*-gentiobioside (**4**), 86.31 μg∙mL^−1^ trachelogenin 4'-*O*-*β*-gentiobioside (**5**), 91.60 μg∙mL^−1^ matairesinoside (**6**), 280.56 μg∙mL^−1^ tracheloside (**7**), 107.38 μg∙mL^−1^ arctigenin 4'-*O*-*β*-gentiobioside (**8**), 61.56 μg∙mL^−1^ nortrachelogenin (**9**), 86.40 μg∙mL^−1^ arctiin (**10**), 49.50 μg∙mL^−1^ matairesinol (**11**), 125.20 μg∙mL^−1^ trachelogenin (**12**), 12.35 μg∙mL^−1^ 5-methoxytrachelogenin (**13**), 22.76 μg∙mL^−1^ arctigenin (**14**). Meanwhile, a mixed solution including above solution and 58.46 μg∙mL^−1^ tanegoside A (**17**) was prepared for qualitative analysis. An aliquot of 10 μL was injected for HPLC-UV analysis and 1 μL for HPLC-QTOF-MS analysis. All the solutions were stored at 4 °C and brought to room temperature before use.

### 3.3. Preparation of Caulis Trachelospermi for HPLC-UV Analysis 

Fourteen batches of Caulis Trachelospermi samples were pulverized and passed through a 100 mesh screen. Four hundred milligrams of the obtained fine powder was accurately weighed into a 50 mL capped conical flask, and 20 mL 50% aqueous methanol was accurately added. Sonication was performed at room temperature for 30 min, and then the same solvent was added to compensate for the lost weight during the extraction. The extracts were filtered with a 0.45 μm membrane filter prior to HPLC analysis, discarding the first part of the filtrate. An aliquot of 20 μL was injected for HPLC-UV analysis.

### 3.4. Preparation of the Sample of Caulis Trachelospermi for HPLC-QTOF-MS Analysis 

Caulis Trachelospermi (Batch no. 080130, 160 g) was extracted two times with 80% alcohol at boiling temperature. The extract was concentrated and diluted in 1600 mL 5% alcohol. The solution was first centrifuged to remove the insoluble substance and then was passed through a HP-20 macroporous resin column (100 mL) and eluted by 500 mL water and 500 mL 70% alcohol successively. The 70% alcohol elution was concentrated and dried to produce 5.3 g product. 10.0 mg of the product weighed accurately was dissolved into a 10 mL volumetric flask and adjusted to volume with methanol-water (50:50, v/v) to obtain the sample solution. Prior to injection, the solution was passed through a 0.45 μm membrane filter. An aliquot of 1 μL was injected for HPLC-QTOF-MS analysis.

### 3.5. HPLC-UV Conditions for Quantitative Analysis 

Quantitative analysis was performed on an Agilent 1200 series HPLC-UV system (Agilent Technologies, Santa Clara, CA, USA), compressing a quaternary pump, a vacuum degasser, an autosampler, a thermostatted column compartment and a UV-vis detector. Separation was done on an Agilent Zorbax Eclipse Plus C18 column (4.6 mm × 150 mm, 5 μm) and column temperature was maintained at 30 °C. The mobile phase was water (A) and methanol (B) with a linear gradient program as follows: 0–15 min, 10%–30% B; 15–40 min, 30%–40% B; and 40–60 min, 40%–60% B. Re-equilibration duration was 30 min between individual runs and the flow rate was kept at 0.8 mL∙min^-1^. The detector wavelength was set at 230 nm [19].

### 3.6. HPLC-QTOF-MS Conditions for Qualitative Analysis 

Chromatography was performed using a Waters ACQUITY UHPLC system (Waters Corporation, Milford, MA, USA), equipped with a binary solvent delivery system and an autosampler. HPLC conditions were the same as those for quantitative analysis.

The Waters ACQUITY XEVO G2 QTOF mass spectrometer (Waters Corporation, Manchester, UK) was interfaced to the UHPLC system via an electrospray ionization (ESI) source. The source was operated in positive ionization mode. The desolvation gas was set to 600 L∙h^−1^ at temperature of 300 °C, the cone gas set to 50 L∙h^−1^, and the source temperature set to 100 °C. The capillary voltage and cone voltage were set to 3000 V and 20 V, respectively. The TOF data were collected between *m*/*z* 50 and 1200. The MS/MS experiments were performed using variable collision energy (20–30 eV). The accurate mass and composition for the precursor and fragment ions were calculated using Masslynx 4.1 software (Waters Corp., Milford, MA, USA) that was incorporated in the instrument.

### 3.7. Validation of the Quantitative Method 

#### 3.7.1. Calibration Curve, Limits of Detection and Quantification

For the calibration curves, a 1, 2, 3, 5, 10, 15, 20, 25, 35 μL volume of the mixed standard solution was injected respectively, and then the calibration curves were constructed by plotting the peak area *versus* the concentration (ng) of each analyte. The limit of detection (LOD) and the limit of quantification (LOQ) under the present chromatographic conditions were determined by injecting a series of diluted standard solutions when the signal-to-noise ratio (S/N) of analytes were about 3 and 10, respectively.

#### 3.7.2. Precision and Accuracy

Precision of the developed method was evaluated in six replicates of the mixed standard solutions within one and three consecutive days to determine intra and inter-day precision, respectively. Variations of the peak area were taken as the measures of precision and expressed as relative standard deviation (RSD).

Recovery test was used to evaluate the accuracy of this method. The test was performed by adding accurate amounts of the mixed standard solutions into 200 mg of Caulis Trachelospermi (Batch no. 080130) in sextuplicate. The mixture were then extracted and analyzed as described in Section 3.3 and Section 3.5. The average recovery percentage was calculated by the formula: recovery (%) = (observed amount − original amount)/spiked amount × 100%.

#### 3.7.3. Repeatability and Stability

To confirm the repeatability, six independent samples were prepared and analyzed from the same sample (Batch no. 080130). Stability was assessed through analyzing replicate injections of the same sample at 0 h, 2 h, 4 h, 6 h, 8 h and 24 h, which were stored at 25 °C. The relative standard deviation (RSD) was used to evaluate the results.

## 4. Conclusions

In the present study, a HPLC-UV method was first developed for the simultaneous determination of 14 dibenzylbutyrolatone lignans in Caulis Trachelospermi. The developed method was validated for all parameters and has been successfully applied to analyze 14 batches Caulis Trachelospermi samples, which could be helpful in quality assessment and standardization of Caulis Trachelospermi and its product. Meanwhile, a HPLC-QTOF-MS method was employed for the identification and structural characterization of major constituents in the sample extracted from Caulis Trachelospermi. The specific fragment ions obtained by MS/MS provide sufficient information for structure elucidation. Moreover, the fragmentation patterns of lignano-8'-hydroxy-9, 9'-lactones were investigated for the first time in this work. This qualitative identification method provide essential data for further chemical or pharmacological studies of Caulis Trachelospermi, and may be applied for the identification of bioactive dibenzylbutyrolatone lignans from other related plants.

## Supplementary Materials

Supplementary materials can be accessed at: http://www.mdpi.com/1420-3049/20/05/8107/s1.

## References

[1] Committee of pharmacopoeia of the People’s Republic of China (2010). Pharmacopoeia of the People’s Republic of China (First Part).

[2] Nishibe S., Han Y.M. (2002). Chemical constituents from *Trachelosperomum jasminoides* and its anticancer activity. World Phytomed..

[3] Lee M.H., Lee J.M., Jun S.H., Ha C.G., Lee S.-H., Kim N.M., Lee J.H., Ko N.Y., Mun S.H., Park S.H. (2007). *In vitro* and *in vivo* anti-inflammatory action of the ethanol extract of Trachelospermi Caulis. J. Pharm. Pharmacol..

[4] Tan X.Q., Chen H.S., Liu R.H., Tan C.H., Xu C.L., Xuan W.D., Zhang W.D. (2005). Lignans from *Trachelospermum jasminoides*. Planta Med..

[5] Tan X.Q., Chen H.S., Zhou M., Zhang Y. (2006). Triterpenoids from canes with leaves of *Trachelospermum jasminoides*. Chin. Tradit. Herb. Drugs.

[6] Tan X.Q., Guo L.J., Chen H.S., Wu L.S., Kong F.F. (2010). Study on the flavonoids constituents of *Trachelospermum jasminoides*. J. Chin. Med. Mater..

[7] Jing L., Yu N.J., Li Y.S., Fu L., Zhao Y.M. (2011). Novel lignans from the stems and leaves of *Trachelospermum jasminoides*. Chin. Chem. Lett..

[8] Zhu C.C., Jing L., Yu N.J., Yang X.D., Zhao Y.M. (2013). A new lignan and active compounds inhibiting NF-*κ*B signaling pathway from Caulis Trachelospermi. Acta Pharm. Sin. B.

[9] Liu X.T., Wang Z.X., Yang Y., Wang L., Sun R.F., Zhao Y.M., Yu N.J. (2014). Active components with inhibitory activities on IFN-γ/STAT1 and IL-6/STAT3 signaling pathways from Caulis Trachelospermi. Molecules.

[10] Li X.X., Wan L.L., Zhu J.H., Li Y., Zhang J.P., Guo C. (2008). Determination of flavonoid aglycones in *Trachelospermum jasminoides* (Lindl.) Lem by HPLC. China Pharm..

[11] Tan X.Q., Guo L.J., Kong F.F. (2011). Determination of trachelogenin in *Trachelospermum jasminoides* (*Lindl*.) Lem. by HPLC. Anhui Med. Pharm. J..

[12] Guo L., Tan X., Lu P., Wu L., Kong F. (2009). Determination of salicylic acid in *Trachelospermum jasminoides* (*Lindl*.) Lem. by HPLC. China Pharm..

[13] Kong M., Zhang J., Yao N., Li Y., Jiang C.H., Gao M., Fang Z.J., Liang J.Y. (2013). Simultaneous determination of five compounds in *Trachelospermum jasminodes* by HPLC. Chin. J. Exp. Tradit. Med. Formulae.

[14] Zhu S., Tan X., Chen H., Lei Y. (2005). Determination the content of tracheloside in *Trachelospermum jasminoides* (*Lindl*.) Lem. by RP-HPLC. China Pharm..

[15] Fujimoto T., Nose M., Takeda T., Ogihara Y., Nishibe S. (1993). Quantitative analysis of lignan components in Chinese crude drugs “Zihualuoshi” and “Luoshiteng”. Jpn. J. Pharmacogn..

[16] Li Q., Wang Z.M., Fu X.T. (2010). Study on quality standards of trachelospermum jasminoides. Northwest Pharm. J..

[17] Liu M.P., Yu N.J., Zhao J., Xu B., Zhao Y.M. (2010). Quantitative Analysis of Total Lignans in the Lignan Extract from Trachelospermum jasminoides by Ultraviolet Spectrophotomey. Med. Pharm. J. Chin. PLA.

[18] Zhou Q., Fu H.Y., Bai J.W., Li L. (2007). Determination of the Total Flavonoids in Chinese Starjasmine Stem by Spectrophotometry. Lishizhen Med. Mater. Med. Res..

[19] Liu Y.Q., Yu N.J., Yang X.D., Zhao Y.M. (2009). Study on HPLC fingerprint of *Trachelospermum jasminoides*. China J. Chin. Mater. Med..

[20] Ferrer I., García-Reyes J.F., Mezcua M., Thurman E.M., Fernández-Alba A.R. (2005). Multi-residue pesticide analysis in fruits and vegetables by liquid chromatography-time-of-flight mass spectrometry. J. Chromatogr. A.

[21] Xu S.Y., Ye M.L., Xu D.K., Li X., Pan C.S., Zou H.F. (2006). Matrix with high salt tolerance for the analysis of peptide and protein samples by desorption/ionization time-of-flight mass spectrometry. Anal. Chem..

[22] Zhang J.L., Li P., Li H.J., Jiang Y., Ren M.T., Liu Y. (2008). Development and validation of a liquid chromatography/electrospray ionization time-of-flight mass spectrometry method for relative and absolute quantification of steroidal alkaloids in Fritillaria species. J. Chromatogr. A.

[23] Wu H., Guo J., Chen S., Liu X., Zhou Y., Zhang X., Xu X. (2013). Recent developments in qualitative and quantitative analysis of phytochemical constituents and their metabolites using liquid chromatography-mass spectrometry. J. Pharm. Biomed. Anal..

[24] Schmidt T.J., Alfermann A.W., Fuss E. (2008). High-performance liquid chromatography/mass spectrometric identification of dibenzylbutyrolactone type lignans: insights into electrospray ionization tandem mass spectrometric fragmentation of lign-7-eno-9,9'-lactones and application to the lignans of *Linum usitatissimum* L. (Common Flax). Rapid Commun. Mass Spectrom..

[25] Schmidt T.J., Hemmati S., Fuss E., Alfermann A.W. (2006). A combined HPLC-UV and HPLC-MS method for the identification of lignans and its application to the lignans of *Linum usitatissimum* L. and *L. bienne* mill. Phytochem. Anal..

[26] Jing L., Yu N.J., Zhao Y.M., Li Y.S. (2012). Trace chemical constituents contained in *Trachelospermum jasminoides* and structure identification. China J. Chin. Mater. Med..

[27] Yuan Q.S., Yu N.J., Zhao Y.M., Xu B., Yao Z.W. (2010). Chemical constituents from *Trachelospermum jasminoides*. Chin. Tradit. Herb. Drugs.

[28] Yu N.J., Zhao Y.M., Ren F.X. (2005). Total Lignans Extract from Caulis Trachelospermi, Its Extraction Method, and the Medicinal Usage of the Extract and Its Active Constituents.

