# Supplementary material for: Qualitative and Quantitative Analysis of Lignan Constituents in Caulis Trachelospermi by HPLC-QTOF-MS and HPLC-UV
Source: Molecules. 2015 May 5;20(5):8107–24. doi: 10.3390/molecules20058107 (PMC6272670; doi:10.3390/molecules20058107)
Supplement: Supplementary file 1 [file molecules-20-08107-s001.pdf]

Supplementary Materials

Table S1. MS/MS data and proposed fragmentation pathways of reference compounds.

Peak No.	Compounds	t_R (min)	Precursor Ion (*m/z*)	Error (ppm)	Formula	Fragments (*m/z*)	Elem. comp.	Pathways
1	nortrachelogenin 5'-*C*-*β*-D-glucoside	20.090	559.1801 $[M+Na]^+$	1.79	$C_{26}H_{32}O_{12}$	483.1670	$C_{26}H_{27}O_9^+$	$[M+H-3H_2O]^+$
						441.1555	$C_{24}H_{25}O_8^+$	$[M+H-2H_2O-(CHO-CH_2OH)]^+$
						465.1556	$C_{26}H_{25}O_8^+$	$[M+H-4H_2O]^+$
						423.1459	$C_{24}H_{23}O_7^+$	$[M+H-3H_2O-(CHO-CH_2OH)]^+$
						203.0711	$C_{12}H_{11}O_3^+$	$[C+H-HCOH]^+$
						137.0607	$C_8H_9O_2^+$	$[A']^+$
2	nortracheloside	24.370	559.1769 $[M+Na]^+$	−3.93	$C_{26}H_{32}O_{12}$	397.1255	$C_{20}H_{22}O_7Na^+$	$[M+Na-Glc]^+$
						375.1438	$C_{20}H_{23}O_7^+$	$[M+H-Glc]^+$
						357.1332	$C_{20}H_{21}O_6^+$	$[M+H-Glc-H_2O]^+$
						329.1388	$C_{19}H_{21}O_5^+$	$[M+H-Glc-H_2O-CO]^+$
						311.1281	$C_{19}H_{19}O_4^+$	$[M+H-Glc-2H_2O-CO]^+$
						233.0816	$C_{13}H_{13}O_4^+$	$[C+H]^+$
						189.0924	$C_{12}H_{13}O_2^+$	$[C+H-CO_2]^+$
						137.0610	$C_8H_9O_2^+$	$[A]^+/[A']^+$
3	nortrachelogenin 8'-*O*-*β*-D-glucoside	25.227	559.1805 $[M+Na]^+$	2.50	$C_{26}H_{32}O_{12}$	375.1446	$C_{20}H_{23}O_7^+$	$[M+H-Glc]^+$
						357.1342	$C_{20}H_{21}O_6^+$	$[M+H-Glc-H_2O]^+$
						329.1390	$C_{19}H_{21}O_5^+$	$[M+H-Glc-H_2O-CO]^+$
						311.1281	$C_{19}H_{19}O_4^+$	$[M+H-Glc-2H_2O-CO]^+$
						233.0812	$C_{13}H_{13}O_4^+$	$[C+H]^+$
						189.0915	$C_{12}H_{13}O_2^+$	$[C+H-CO_2]^+$
						137.0602	$C_8H_9O_2^+$	$[A]^+/[A']^+$
4	matairesinol 4'-*O*-*β*-gentiobioside	26.719	705.2364 $[M+Na]^+$	−0.99	$C_{32}H_{42}O_{16}$	543.1831	$C_{26}H_{32}O_{11}Na^+$	$[M+Na-Glc]^+$
						381.1292	$C_{20}H_{22}O_6Na^+$	$[M+Na-2Glc]^+$
						159.0422	$C_8H_8O_2Na^+$	$[A+Na]^+/[A'+Na]^+$
						137.0603	$C_8H_9O_2^+$	$[A]^+/[A']^+$

Table S1. *Cont.*

Peak No.	Compounds	t_R (min)	Precursor Ion (*m/z*)	Error (ppm)	Formula	Fragments (*m/z*)	Elem. comp.	Pathways
5	trachelogenin 4'-*O*-*β*-gentiobioside	29.039	730.2925 $[M+NH_4]^+$	0.41	$C_{33}H_{44}O_{17}$	551.2136	$C_{27}H_{35}O_{12}^+$	$[M+H-Glc]^+$
						389.1615	$C_{21}H_{25}O_7^+$	$[M+H-2Glc]^+$
						371.1508	$C_{21}H_{23}O_6^+$	$[M+H-2Glc-H_2O]^+$
						343.1562	$C_{20}H_{23}O_5^+$	$[M+H-2Glc-H_2O-CO]^+$
						325.1445	$C_{20}H_{21}O_4^+$	$[M+H-2Glc-2H_2O-CO]^+$
						247.0982	$C_{14}H_{15}O_4^+$	$[C+H]^+$
						203.1082	$C_{13}H_{15}O_2^+$	$[C+H-CO_2]^+$
						151.0775	$C_9H_{11}O_2^+$	$[A']^+$
						137.0619	$C_8H_9O_2^+$	$[A]^+$
6	matairesinoside	32.188	543.1857 $[M+Na]^+$	2.76	$C_{26}H_{32}O_{11}$	359.1500	$C_{20}H_{23}O_6^+$	$[M+H-Glc]^+$
						341.1393	$C_{20}H_{21}O_5^+$	$[M+H-Glc -H_2O]^+$
						323.1289	$C_{20}H_{19}O_4^+$	$[M+H-Glc -2H_2O]^+$
						223.0973	$C_{12}H_{15}O_4^+$	$[M+H-Glc-A]^+$
						163.0755	$C_{10}H_{11}O_2^+$	$[B]^+$
						137.0599	$C_8H_9O_2^+$	$[A]^+/[A']^+$
7	tracheloside	33.679	573.1951 $[M+Na]^+$	0.52	$C_{27}H_{34}O_{12}$	389.1603	$C_{21}H_{25}O_7^+$	$[M+H-Glc]^+$
						371.1501	$C_{21}H_{23}O_6^+$	$[M+H-Glc-H_2O]^+$
						343.1548	$C_{20}H_{23}O_5^+$	$[M+H-Glc-H_2O-CO]^+$
						325.1444	$C_{20}H_{21}O_4^+$	$[M+H-Glc-2H_2O-CO]^+$
						247.0973	$C_{14}H_{15}O_4^+$	$[C+H]^+$
						203.1075	$C_{13}H_{15}O_2^+$	$[C+H-CO_2]^+$
						151.0763	$C_9H_{11}O_2^+$	$[A']^+$
						137.0605	$C_8H_9O_2^+$	$[A]^+$

Table S1. *Cont.*

Peak No.	Compounds	t_R (min)	Precursor Ion (*m/z*)	Error (ppm)	Formula	Fragments (*m/z*)	Elem. comp.	Pathways
8	arctigenin 4'-*O*-*β*-gentiobioside	34.517	714.2961 $[M+NH_4]^+$	−1.68	$C_{33}H_{44}O_{16}$	373.1645	$C_{21}H_{25}O_6^+$	$[M+H-2Glc]^+$
						355.1540	$C_{21}H_{23}O_5^+$	$[M+H-2Glc-H_2O]^+$
						237.1117	$C_{13}H_{17}O_4^+$	$[M+H-2Glc-A]^+$
						137.0601	$C_8H_9O_2^+$	$[A]^+$
9	nortrachelogenin	37.508	397.1262 $[M+Na]^+$	−0.25	$C_{20}H_{22}O_7$	357.1342	$C_{20}H_{21}O_6^+$	$[M+H-H_2O]^+$
						329.1392	$C_{19}H_{21}O_5^+$	$[M+H-H_2O-CO]^+$
						311.1288	$C_{19}H_{19}O_4^+$	$[M+H-2H_2O-CO]^+$
						233.0822	$C_{13}H_{13}O_4^+$	$[C+H]^+$
						189.0925	$C_{12}H_{13}O_2^+$	$[C+H-CO_2]^+$
						175.0766	$C_{11}H_{11}O_2^+$	$[C+H-CO-HCOH]^+$
						137.0614	$C_8H_9O_2^+$	$[A]^+/[A']^+$
10	arctiin	40.640	557.1993 $[M+Na]^+$	−1.08	$C_{27}H_{34}O_{11}$	373.1649	$C_{21}H_{25}O_6^+$	$[M+H-Glc]^+$
						355.1544	$C_{21}H_{23}O_5^+$	$[M+H-Glc-H_2O]^+$
						337.1439	$C_{21}H_{21}O_4^+$	$[M+H-Glc-2H_2O]^+$
						295.1338	$C_{19}H_{19}O_3^+$	$[M+H-Glc-H_2O-2HCOH]^+$
						237.1127	$C_{13}H_{17}O_4^+$	$[M+H-Glc-A]^+$
						177.0923	$C_{11}H_{13}O_2^+$	$[B]^+$
						151.0759	$C_9H_{11}O_2^+$	$[A']^+$
						137.0604	$C_8H_9O_2^+$	$[A]^+$
11	matairesinol	46.937	359.1493 $[M+H]^+$	−0.56	$C_{20}H_{22}O_6$	341.1392	$C_{20}H_{21}O_5^+$	$[M+H-H_2O]^+$
						323.1286	$C_{20}H_{19}O_4^+$	$[M+H-2H_2O]^+$
						305.1178	$C_{20}H_{17}O_3^+$	$[M+H-3H_2O]^+$
						291.1019	$C_{19}H_{15}O_3^+$	$[M+H-2H_2O-CH_3OH]^+$
						231.0810	$C_{17}H_{11}O^+$	$[M+H-2H_2O-2CH_3OH-CO]^+$
						223.0968	$C_{12}H_{15}O_4^+$	$[M+H-A]^+$
						163.0762	$C_{10}H_{11}O_2^+$	$[B]^+$
						137.0607	$C_8H_9O_2^+$	$[A]^+/[A']^+$
						131.0503	$C_9H_7O^+$	$[B-CH_3OH]^+$

Table S1. *Cont.*

Peak No.	Compounds	t_R (min)	Precursor Ion (*m/z*)	Error (ppm)	Formula	Fragments (*m/z*)	Elem. comp.	Pathways
12	trachelogenin	48.366	411.1422 $[M+Na]^+$	0.49	$C_{21}H_{24}O_7$	371.1496	$C_{21}H_{23}O_6^+$	$[M+H-H_2O]^+$
						343.1548	$C_{20}H_{23}O_5^+$	$[M+H-H_2O-CO]^+$
						325.1443	$C_{20}H_{21}O_4^+$	$[M+H-2H_2O-CO]^+$
						247.0977	$C_{14}H_{15}O_4^+$	$[C+H]^+$
						203.1082	$C_{13}H_{15}O_2^+$	$[C+H-CO_2]^+$
						189.0918	$C_{12}H_{13}O_2^+$	$[C'+H-CO_2]^+$
						151.0770	$C_9H_{11}O_2^+$	$[A']^+$
						137.0613	$C_8H_9O_2^+$	$[A]^+$
13	5-methoxytrachelogenin	49.403	441.1527 $[M+Na]^+$	0.45	$C_{22}H_{26}O_8$	181.0861	$C_{10}H_{13}O_3^+$	$[A']^+$
						159.0433	$C_8H_8O_2Na^+$	$[A+Na]^+$
						137.0613	$C_8H_9O_2^+$	$[A]^+$
14	arctigenin	53.235	395.1488 $[M+Na]^+$	4.30	$C_{21}H_{24}O_6$	137.0599	$C_8H_9O_2^+$	$[A]^+$
						159.0405	$C_8H_8O_2Na^+$	$[A+Na]^+$

Table S2. Constituents comparing with reference compounds detected in the sample extraced from Caulis Trachelospermi.

Peak No.	t_R (min)	Precursor Ion (*m/z*)	Error (ppm)	Formula	Fragments (*m/z*)	Elem. comp.	Pathways	Identity
1	20.090	559.1797 $[M+Na]^+$	1.07	$C_{26}H_{32}O_{12}$	501.1779	$C_{26}H_{29}O_{10}^+$	$[M+H-2H_2O]^+$	nortrachelogenin 5'-*C*-*β*-D-glucoside
					483.1664	$C_{26}H_{27}O_9^+$	$[M+H-3H_2O]^+$	
					465.1555	$C_{26}H_{27}O_9^+$	$[M+H-4H_2O]^+$	
					441.1555	$C_{24}H_{25}O_8^+$	$[M+H-2H_2O-(CHO-CH_2OH)]^+$	
					423.1448	$C_{24}H_{23}O_7^+$	$[M+H-3H_2O-(CHO-CH_2OH)]^+$	
					399.1450	$C_{22}H_{23}O_7^+$	$[M+H-H_2O-C_4H_6O_3-H_2O]^+$	
					203.0725	$C_{12}H_{11}O_3^+$	$[C+H-HCOH]^+$	
					137.0614	$C_8H_9O_2^+$	$[A']^+$	

Table S2. *Cont.*

Peak No.	t_R (min)	Precursor Ion (*m/z*)	Error (ppm)	Formula	Fragments (*m/z*)	Elem. comp.	Pathways	Identity
					375.1443	$C_{20}H_{23}O_7^+$	$[M+H-Glc]^+$	
					357.1341	$C_{20}H_{21}O_6^+$	$[M+H-Glc-H_2O]^+$	
					329.1397	$C_{19}H_{21}O_5^+$	$[M+H-Glc-H_2O-CO]^+$	
2	24.416	559.1776 $[M+Na]^+$	−2.68	$C_{26}H_{32}O_{12}$	311.1290	$C_{19}H_{19}O_4^+$	$[M+H-Glc-2H_2O-CO]^+$	nortracheloside
					233.0822	$C_{13}H_{13}O_4^+$	$[C+H]^+$	
					189.0927	$C_{12}H_{13}O_2^+$	$[C+H-CO_2]^+$	
					137.0620	$C_8H_9O_2^+$	$[A]^+/[A']^+$	
					375.1459	$C_{20}H_{23}O_7^+$	$[M+H-Glc]^+$	
					357.1356	$C_{20}H_{21}O_6^+$	$[M+H-Glc-H_2O]^+$	
					329.1408	$C_{19}H_{21}O_5^+$	$[M+H-Glc-H_2O-CO]^+$	
3	25.296	559.1802 $[M+Na]^+$	1.97	$C_{26}H_{32}O_{12}$	311.1293	$C_{19}H_{19}O_4^+$	$[M+H-Glc-2H_2O-CO]^+$	nortrachelogenin 8'-*O*-*β*-D-glucoside
					233.0830	$C_{13}H_{13}O_4^+$	$[C+H]^+$	
					189.0930	$C_{12}H_{13}O_2^+$	$[C+H-CO_2]^+$	
					137.0612	$C_8H_9O_2^+$	$[A]^+/[A']^+$	
					543.1852	$C_{26}H_{32}O_{11}Na^+$	$[M+Na-Glc]^+$	
4	26.719	705.2364 $[M+Na]^+$	−0.99	$C_{32}H_{42}O_{16}$	381.1320	$C_{20}H_{22}O_6Na^+$	$[M+Na-2Glc]^+$	matairesinol 4'-*O*-*β*-gentiobioside
					159.0423	$C_8H_8O_2Na^+$	$[A+Na]^+/[A'+Na]^+$	
					137.0620	$C_8H_9O_2^+$	$[A]^+/[A']^+$	
					551.2149	$C_{27}H_{35}O_{12}^+$	$[M+H-Glc]^+$	
					389.1616	$C_{21}H_{25}O_7^+$	$[M+H-2Glc]^+$	
					371.1515	$C_{21}H_{23}O_6^+$	$[M+H-2Glc-H_2O]^+$	
					343.1567	$C_{20}H_{23}O_5^+$	$[M+H-2Glc-H_2O-CO]^+$	
5	29.039	730.2937 $[M+NH_4]^+$	2.05	$C_{33}H_{44}O_{17}$	325.1446	$C_{20}H_{21}O_4^+$	$[M+H-2Glc-2H_2O-CO]^+$	trachelogenin 4'-*O*-*β*-gentiobioside
					247.0984	$C_{14}H_{15}O_4^+$	$[C+H]^+$	
					203.1091	$C_{13}H_{15}O_2^+$	$[C+H-CO_2]^+$	
					151.0773	$C_9H_{11}O_2^+$	$[A']^+$	
					137.0615	$C_8H_9O_2^+$	$[A]^+$	

Table S1. *Cont.*

Peak No.	t_R (min)	Precursor Ion (*m/z*)	Error (ppm)	Formula	Fragments (*m/z*)	Elem. comp.	Pathways	Identity
6	32.188	543.1849 [M+Na]$^+$	1.29	$C_{26}H_{32}O_{11}$	359.1506	$C_{20}H_{23}O_6^+$	[M+H−Glc]$^+$	matairesinoside
					341.1404	$C_{20}H_{21}O_5^+$	[M+H−Glc−H_2O]$^+$	
					323.1299	$C_{20}H_{19}O_4^+$	[M+H−Glc−2H_2O]$^+$	
					223.0980	$C_{12}H_{15}O_4^+$	[M+H−Glc−A]$^+$	
					163.0770	$C_{10}H_{11}O_2^+$	[B]$^+$	
					137.0615	$C_8H_9O_2^+$	[A]$^+$/[A']$^+$	
7	33.679	573.1940 [M+Na]$^+$	−1.40	$C_{27}H_{34}O_{12}$	389.1601	$C_{21}H_{25}O_7^+$	[M+H−Glc]$^+$	tracheloside
					371.1499	$C_{21}H_{23}O_6^+$	[M+H−Glc−H_2O]$^+$	
					343.1549	$C_{20}H_{23}O_5^+$	[M+H−Glc−H_2O−CO]$^+$	
					325.1445	$C_{20}H_{21}O_4^+$	[M+H−Glc−2H_2O−CO]$^+$	
					247.0975	$C_{14}H_{15}O_4^+$	[C+H]$^+$	
					203.1078	$C_{13}H_{15}O_2^+$	[C+H−CO_2]$^+$	
					151.0768	$C_9H_{11}O_2^+$	[A']$^+$	
					137.0610	$C_8H_9O_2^+$	[A]$^+$	
8	34.517	714.2968 [M+NH_4]$^+$	−0.70	$C_{33}H_{44}O_{16}$	373.1658	$C_{21}H_{25}O_6^+$	[M+H−2Glc]$^+$	arctigenin 4'-*O*-*β*-gentiobioside
					355.1570	$C_{21}H_{23}O_5^+$	[M+H−2Glc−H_2O]$^+$	
					237.1138	$C_{13}H_{17}O_4^+$	[M+H−2Glc−A]$^+$	
					137.0598	$C_8H_9O_2^+$	[A]$^+$	
9	37.657	397.1275 [M+Na]$^+$	3.02	$C_{20}H_{22}O_7$	357.1343	$C_{20}H_{21}O_6^+$	[M+H−H_2O]$^+$	nortrachelogenin
					329.1394	$C_{19}H_{21}O_5^+$	[M+H−H_2O−CO]$^+$	
					311.1288	$C_{19}H_{19}O_4^+$	[M+H−2H_2O−CO]$^+$	
					233.0819	$C_{13}H_{13}O_4^+$	[C+H]$^+$/[C'+H]$^+$	
					189.0923	$C_{12}H_{13}O_2^+$	[C+H−CO_2]$^+$	
					175.0764	$C_{11}H_{11}O_2^+$	[C+H−CO−HCOH]$^+$	
					137.0609	$C_8H_9O_2^+$	[A]$^+$/[A']$^+$	

Table S2. *Cont.*

Peak No.	t_R (min)	Precursor Ion (*m/z*)	Error (ppm)	Formula	Fragments (*m/z*)	Elem. comp.	Pathways	Identity
10	40.708	557.1996 $[M+Na]^+$	−0.54	$C_{27}H_{34}O_{11}$	373.1651	$C_{21}H_{25}O_6^+$	$[M+H-Glc]^+$	arctiin
					355.1548	$C_{21}H_{23}O_5^+$	$[M+H-Glc-H_2O]^+$	
					337.1438	$C_{21}H_{21}O_4^+$	$[M+H-Glc-2H_2O]^+$	
					295.1339	$C_{19}H_{19}O_3^+$	$[M+H-Glc-H_2O-2HCOH]^+$	
					237.1130	$C_{13}H_{17}O_4^+$	$[M+H-Glc-A]^+$	
					177.0911	$C_{11}H_{13}O_2^+$	$[B]^+$	
					151.0765	$C_9H_{11}O_2^+$	$[A']^+$	
					137.0603	$C_8H_9O_2^+$	$[A]^+$	
11	47.006	359.1510 $[M+H]^+$	4.18	$C_{20}H_{22}O_6$	341.1396	$C_{20}H_{21}O_5^+$	$[M+H-H_2O]^+$	matairesinol
					323.1295	$C_{20}H_{19}O_4^+$	$[M+H-2H_2O]^+$	
					305.1178	$C_{20}H_{17}O_3^+$	$[M+H-3H_2O]^+$	
					291.1021	$C_{19}H_{15}O_3^+$	$[M+H-2H_2O-CH_3OH]^+$	
					263.1074	$C_{18}H_{15}O_2^+$	$[M+H-2H_2O-CO-CH_3OH]^+$	
					231.0814	$C_{17}H_{11}O^+$	$[M+H-2H_2O-2CH_3OH-CO]^+$	
					223.0968	$C_{12}H_{15}O_4^+$	$[M+H-A]^+$	
					163.0758	$C_{10}H_{11}O_2^+$	$[B]^+$	
					137.0603	$C_8H_9O_2^+$	$[A]^+/[A']^+$	
					131.0495	$C_9H_7O^+$	$[B-CH_3OH]^+$	
12	48.446	411.1426 $[M+Na]^+$	1.46	$C_{21}H_{24}O_7$	371.1496	$C_{21}H_{23}O_6^+$	$[M+H-H_2O]^+$	trachelogenin
					343.1549	$C_{20}H_{23}O_5^+$	$[M+H-H_2O-CO]^+$	
					325.1443	$C_{20}H_{21}O_4^+$	$[M+H-2H_2O-CO]^+$	
					247.0970	$C_{14}H_{15}O_4^+$	$[C+H]^+$	
					233.0820	$C_{13}H_{13}O_4^+$	$[C'+H]^+$	
					203.1073	$C_{13}H_{15}O_2^+$	$[C+H-CO_2]^+$	
					189.0912	$C_{12}H_{13}O_2^+$	$[C'+H-CO_2]^+$	
					151.0760	$C_9H_{11}O_2^+$	$[A']^+$	
					137.0604	$C_8H_9O_2^+$	$[A]^+$	

Table S2. *Cont.*

Peak No.	t_R (min)	Precursor Ion (*m/z*)	Error (ppm)	Formula	Fragments (*m/z*)	Elem. comp.	Pathways	Identity
13	48.855	441.1526 $[M+Na]^+$	0.23	$C_{22}H_{26}O_8$				5-methoxytrachelogenin
14	52.955	395.1463 $[M+Na]^+$	−1.01	$C_{21}H_{24}O_6$	159.0408	$C_8H_8O_2Na^+$	$[A+Na]^+$	arctigenin

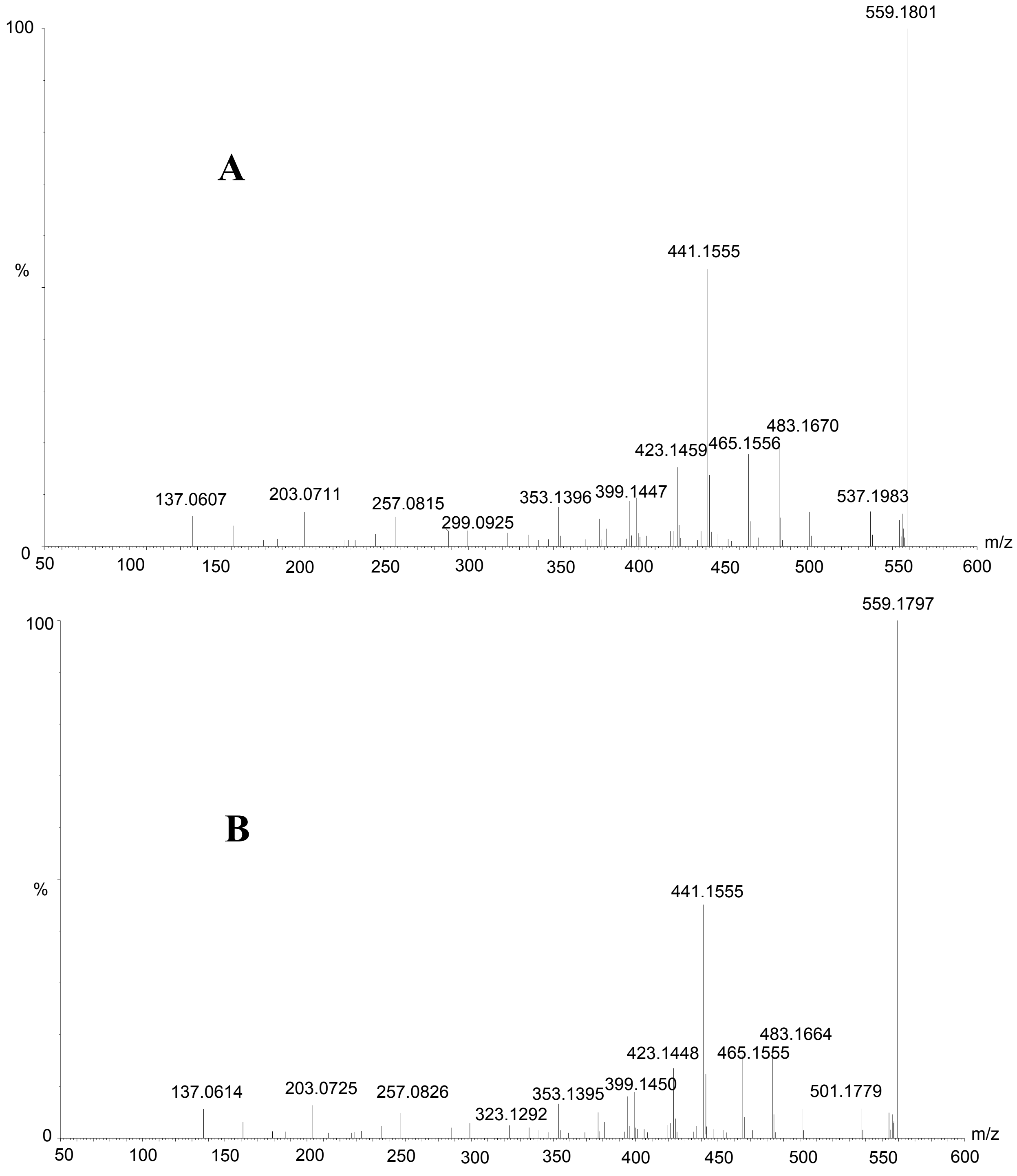

Figure S1. MS/MS spectrum of nortrachelogenin 5'-*C*-*β*-D-glucoside (**1**) in the standard sample (**A**) and in the sample extracted from Caulis Trachelospermi (**B**), respectively.

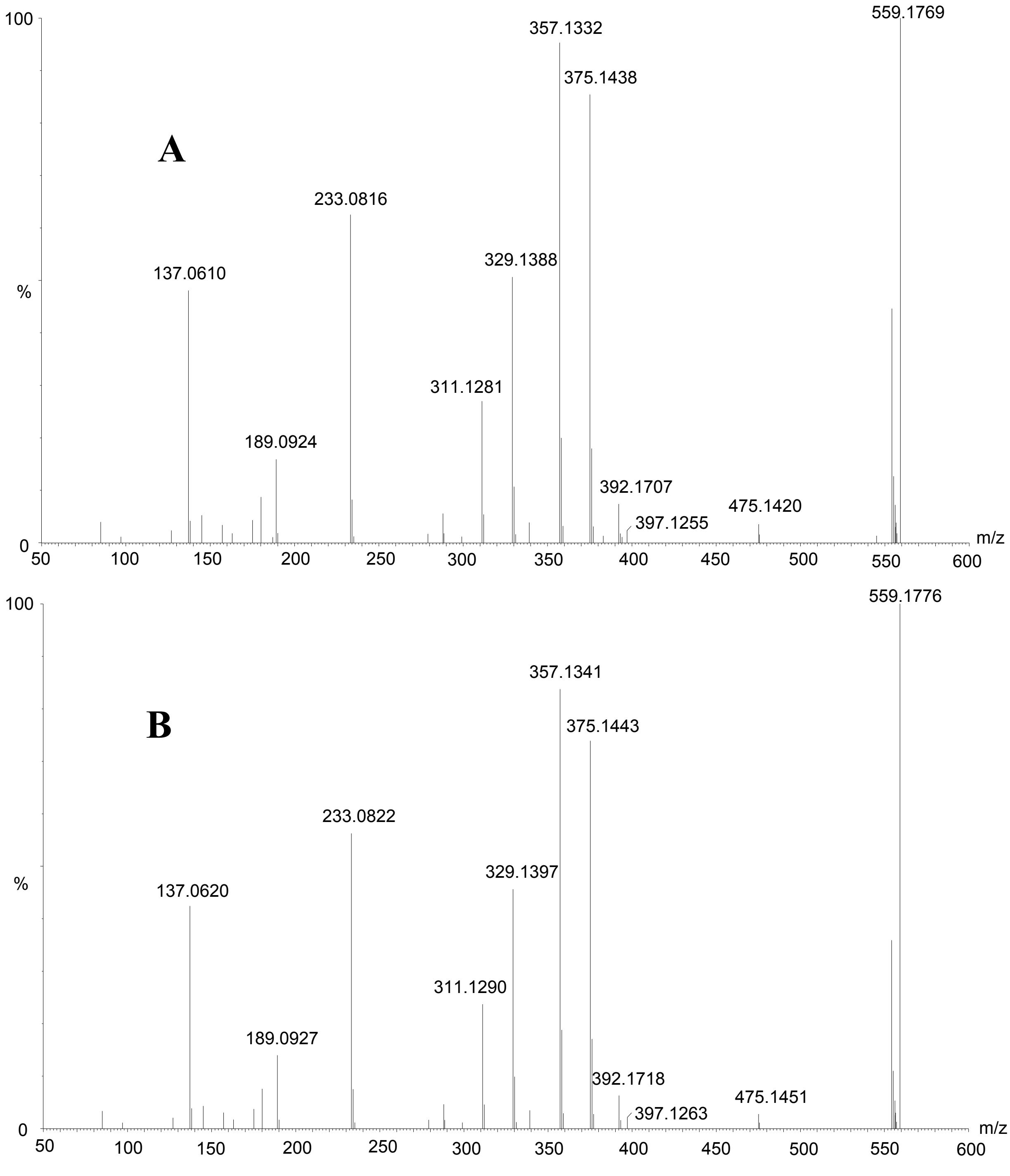

Figure S2. MS/MS spectrum of nortracheloside (**2**) in the standard sample (**A**) and in the sample extracted from Caulis Trachelospermi (**B**), respectively.

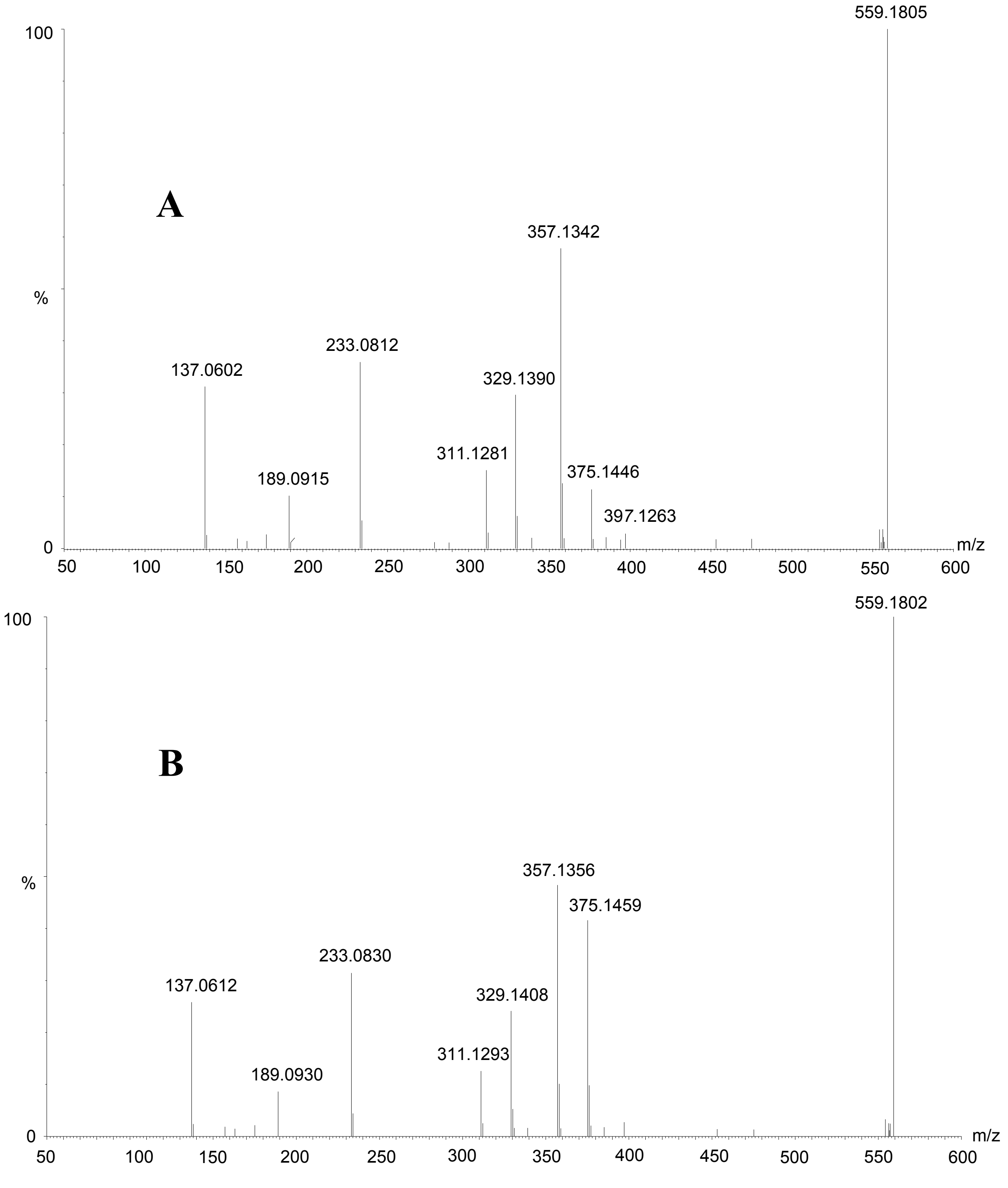

Figure S3. MS/MS spectrum of nortrachelogenin 8'-*O*-*β*-D-glucoside (**3**) in the standard sample (**A**) and in the sample extracted from Caulis Trachelospermi (**B**), respectively.

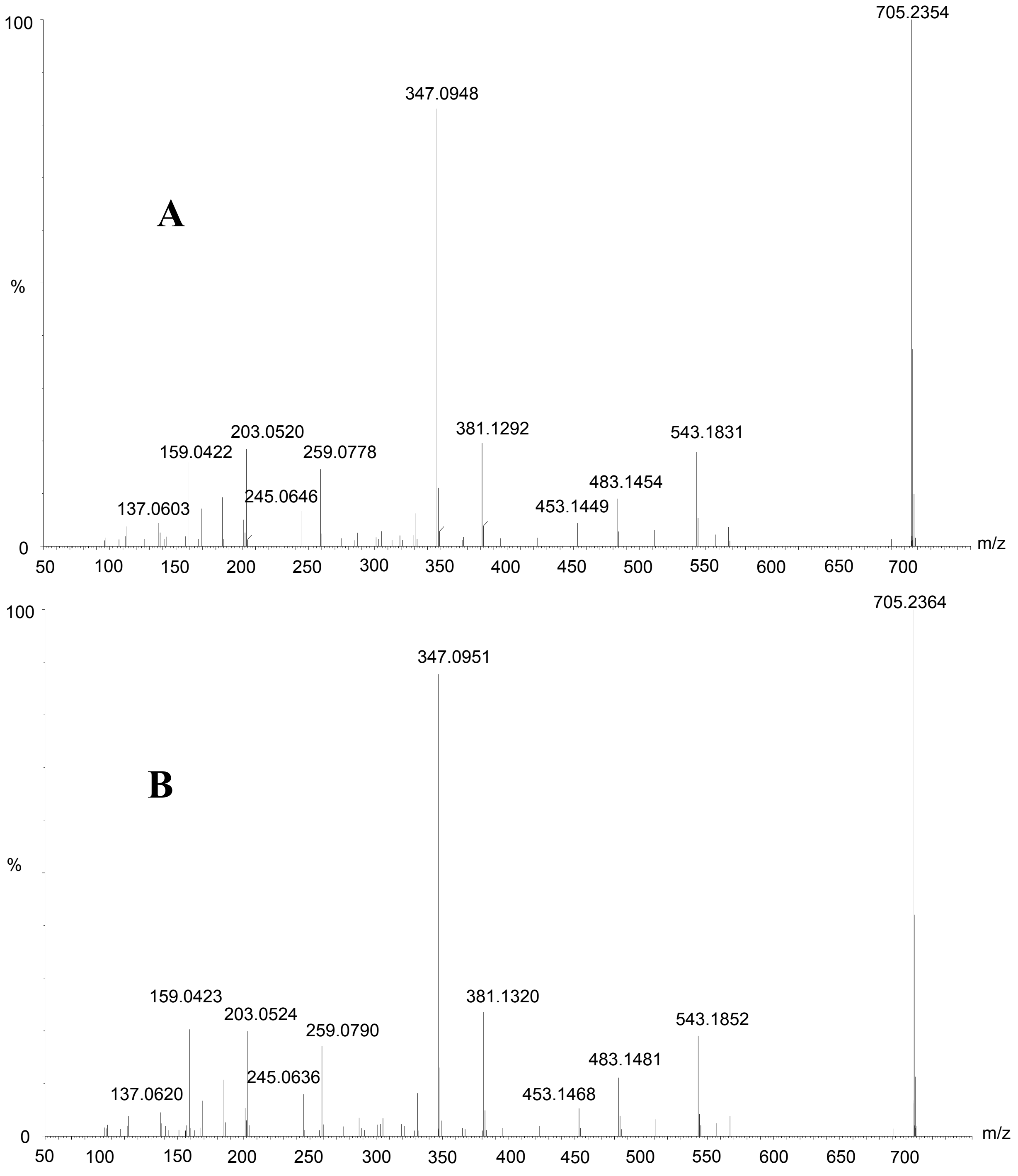

Figure S4. MS/MS spectrum of matairesinol 4'-*O*-*β*-gentiobioside (**4**) in the standard sample (**A**) and in the sample extracted from Caulis Trachelospermi (**B**), respectively.

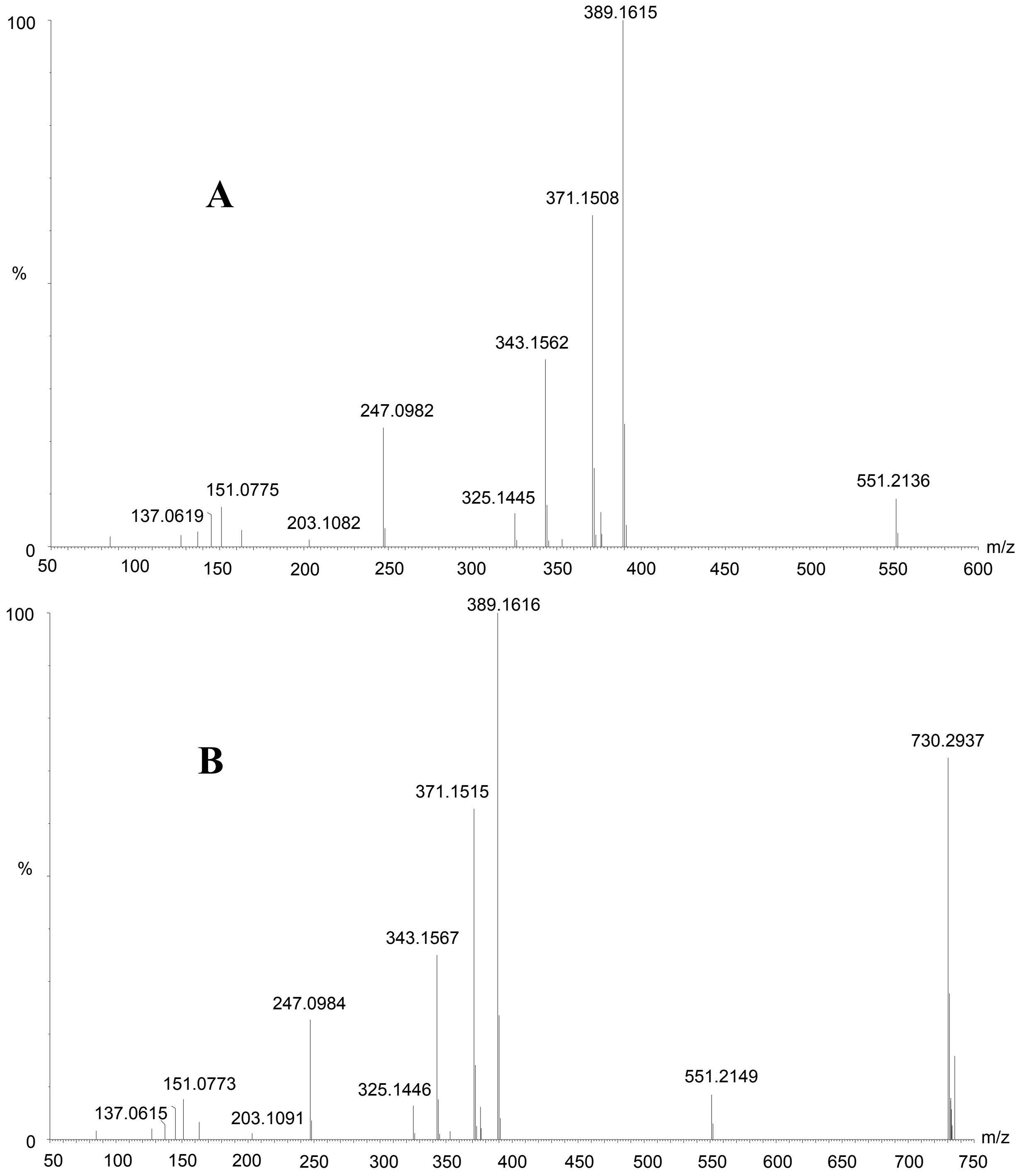

Figure S5. MS/MS spectrum of trachelogenin 4'-*O*-*β*-gentiobioside (**5**) in the standard sample (**A**) and in the sample extracted from Caulis Trachelospermi (**B**), respectively.

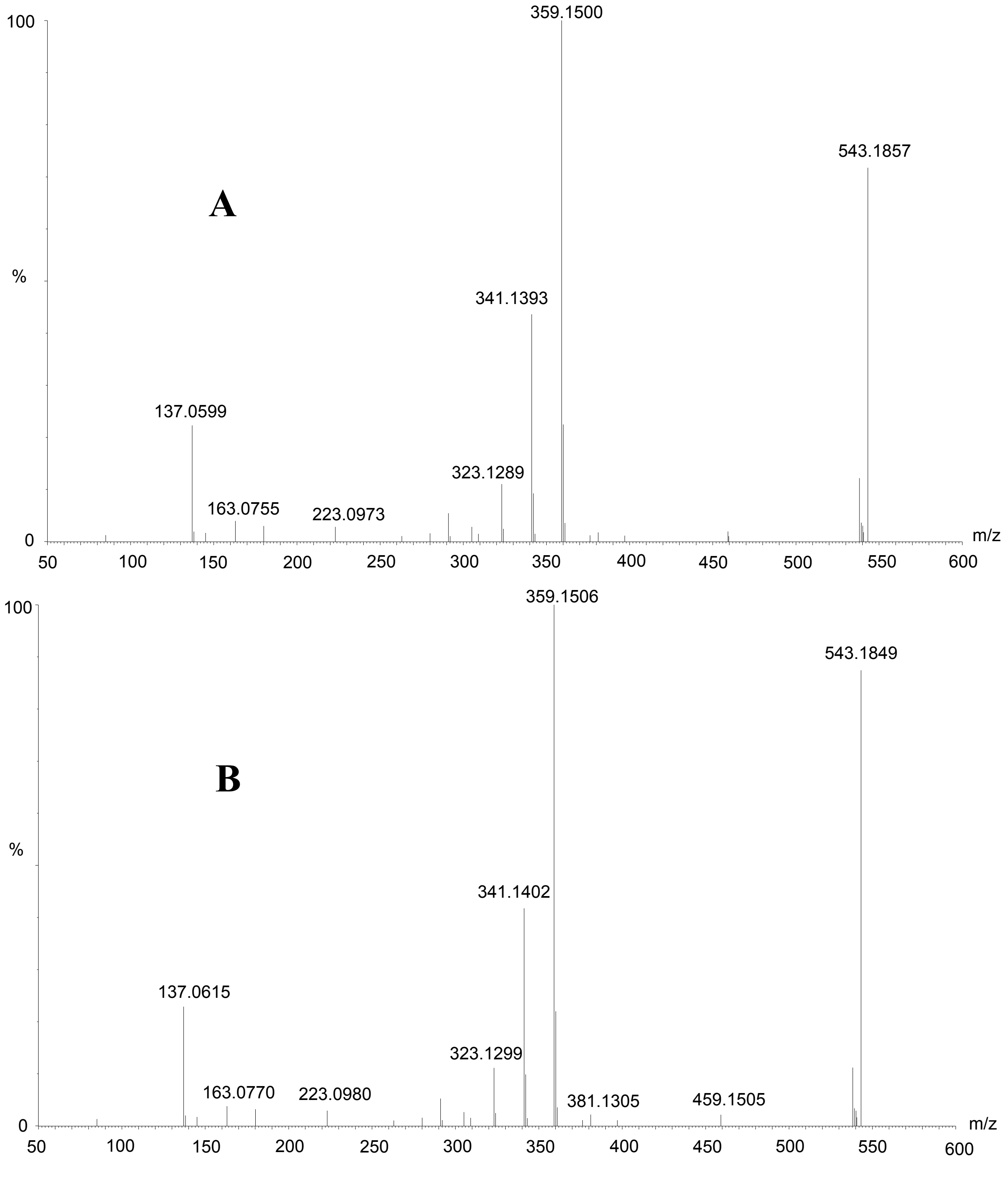

Figure S6. MS/MS spectrum of matairesinoside (**6**) in the standard sample (**A**) and in the sample extracted from Caulis Trachelospermi (**B**), respectively.

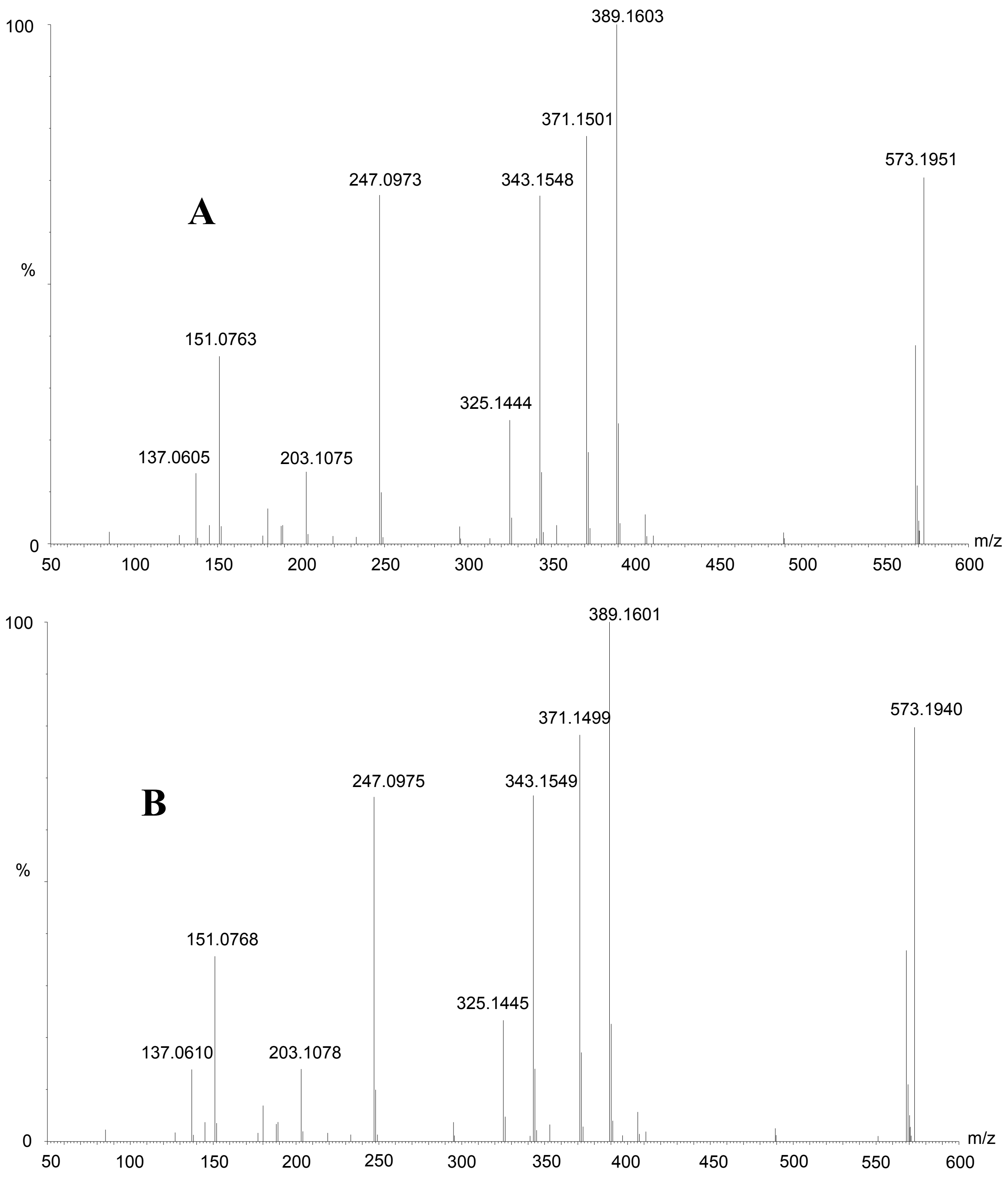

Figure S7. MS/MS spectrum of tracheloside (**7**) in the standard sample (**A**) and in the sample extracted from Caulis Trachelospermi (**B**), respectively.

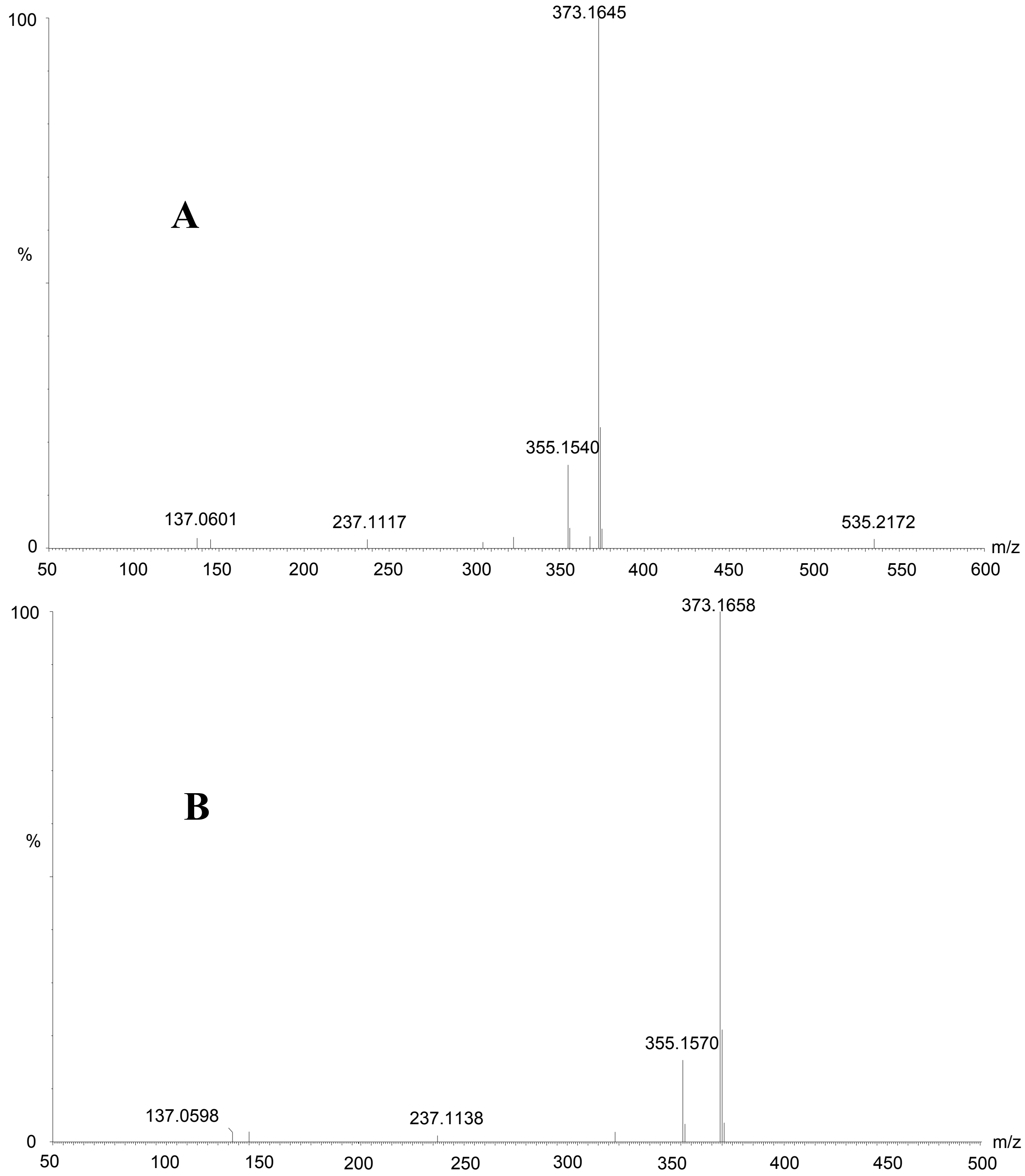

Figure S8. MS/MS spectrum of arctigenin 4'-*O*-*β*-gentiobioside (**8**) in the standard sample (**A**) and in the sample extracted from Caulis Trachelospermi (**B**), respectively.

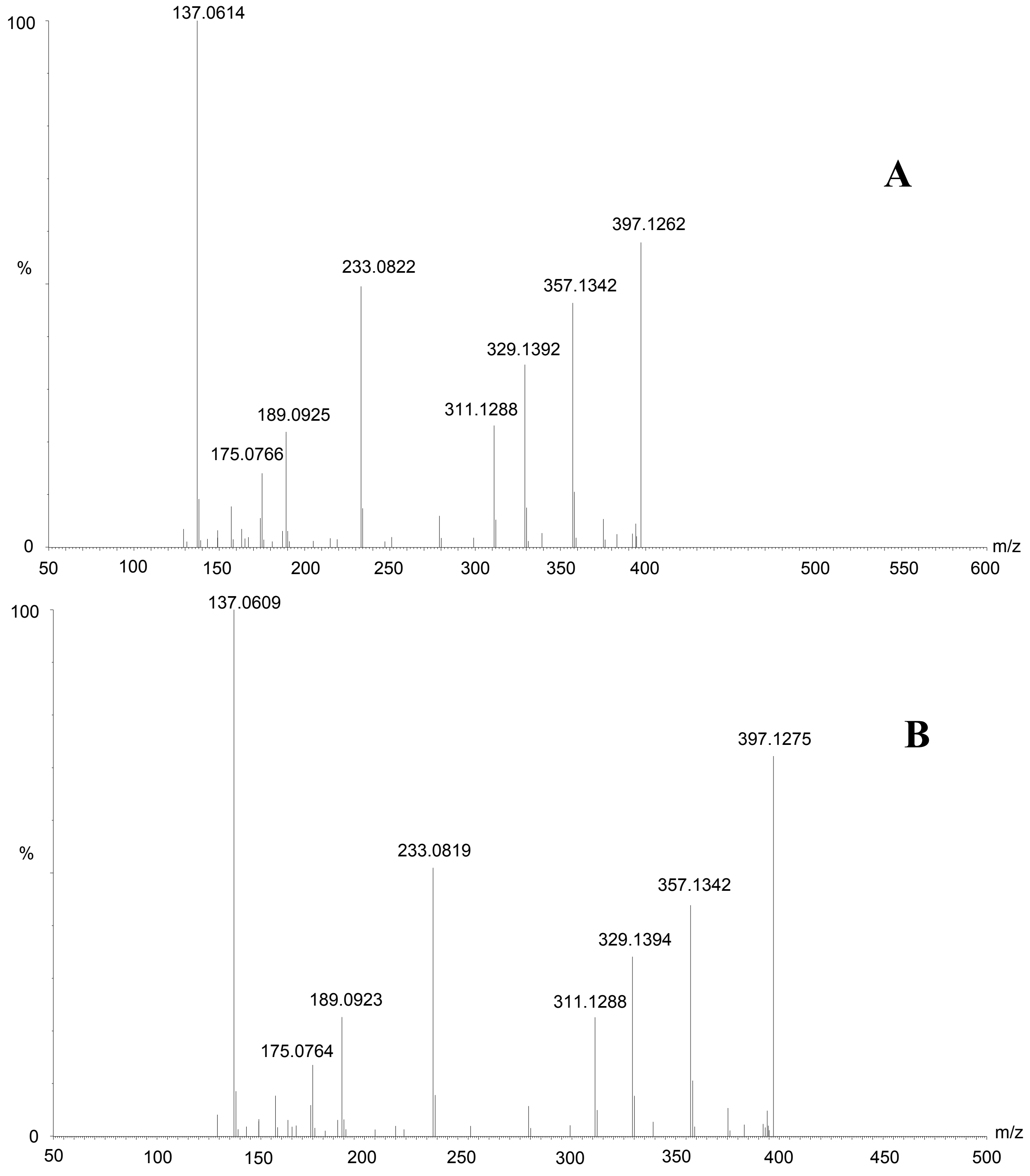

Figure S9. MS/MS spectrum of nortrachelogenin (**9**) in the standard sample (**A**) and in the sample extracted from Caulis Trachelospermi (**B**), respectively.

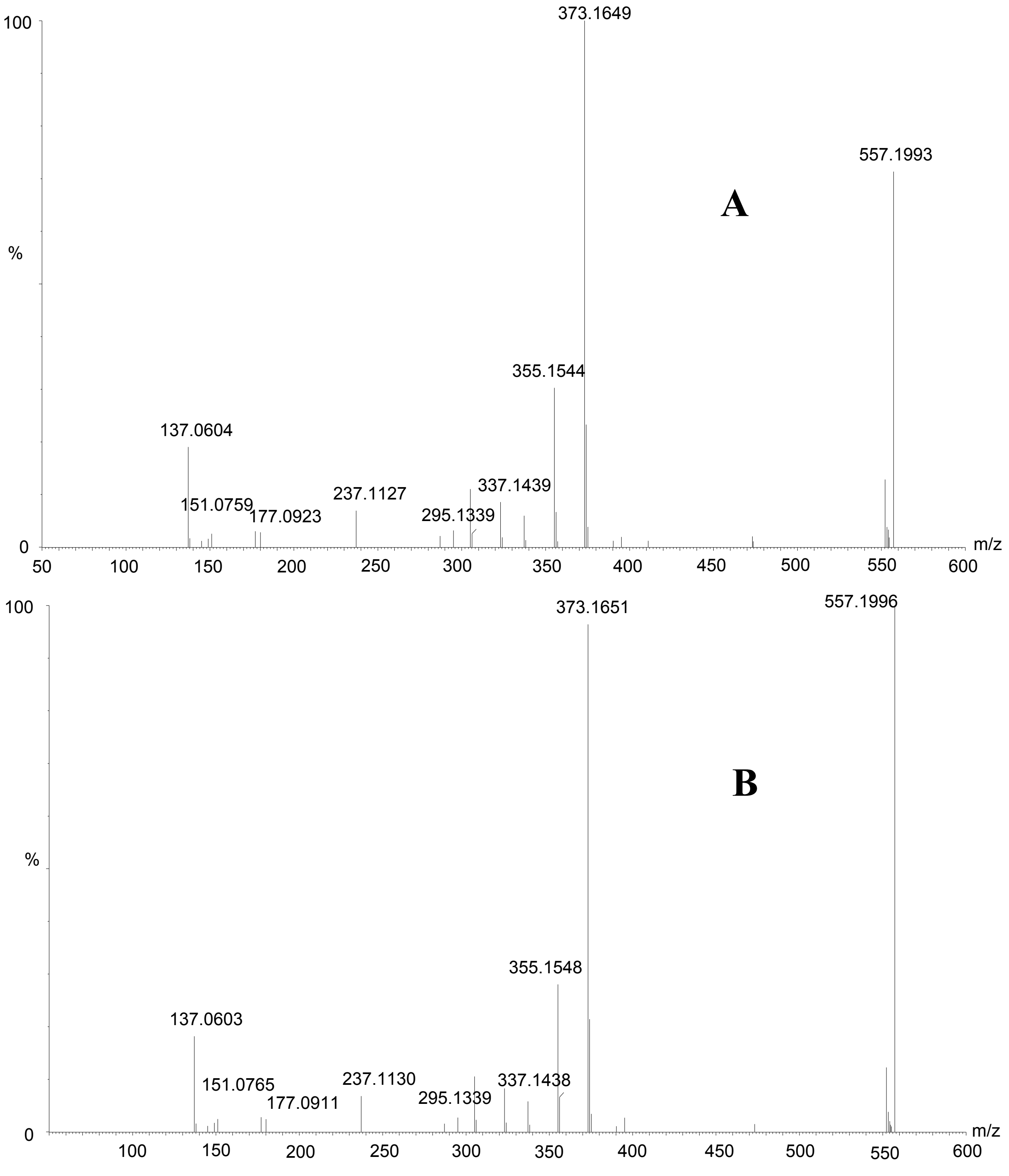

Figure S10. MS/MS spectrum of arctiin (**10**) in the standard sample (**A**) and in the sample extracted from Caulis Trachelospermi (**B**), respectively.

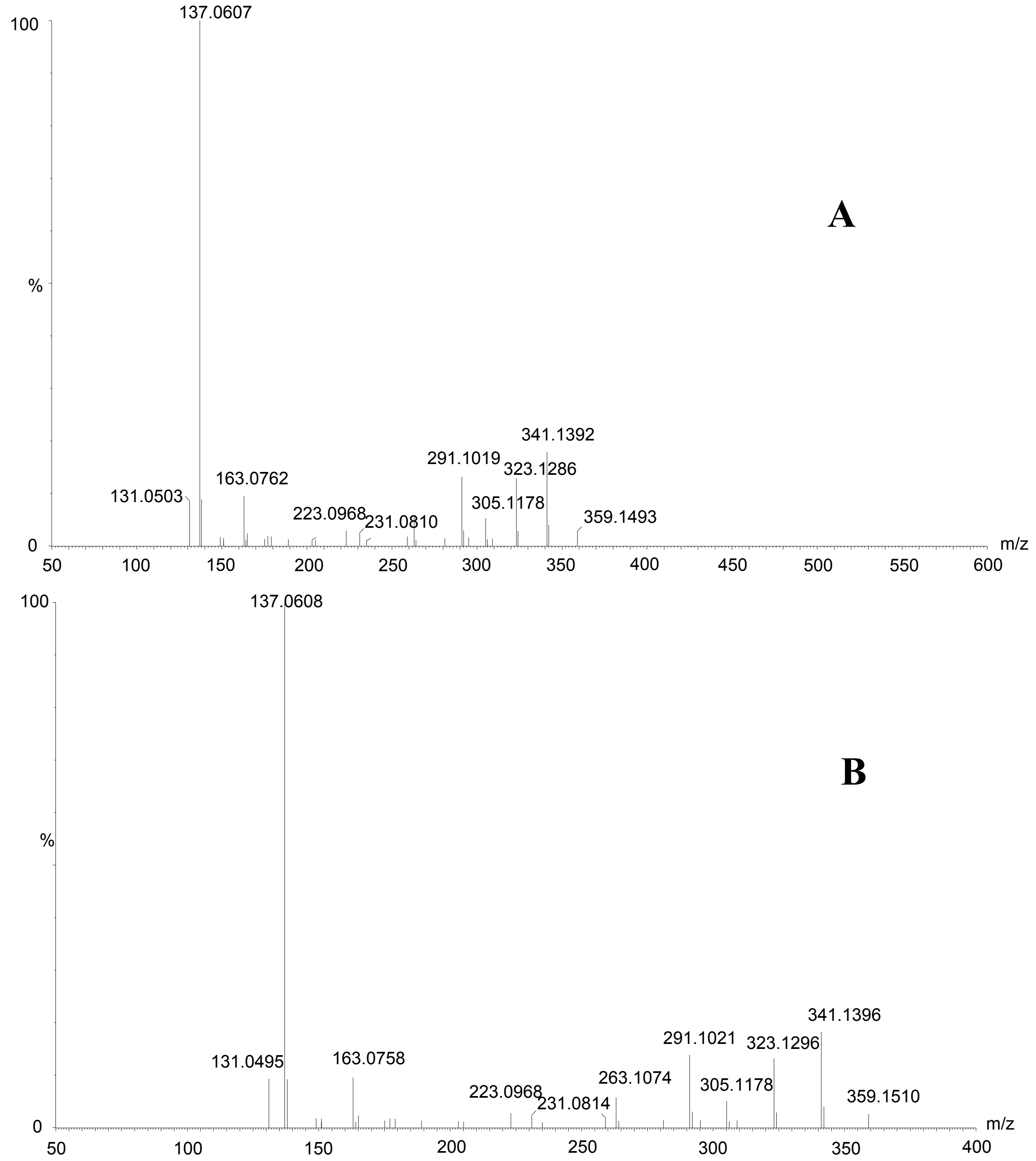

Figure S11. MS/MS spectrum of matairesinol (**11**) in the standard sample (**A**) and in the sample extracted from Caulis Trachelospermi (**B**), respectively.

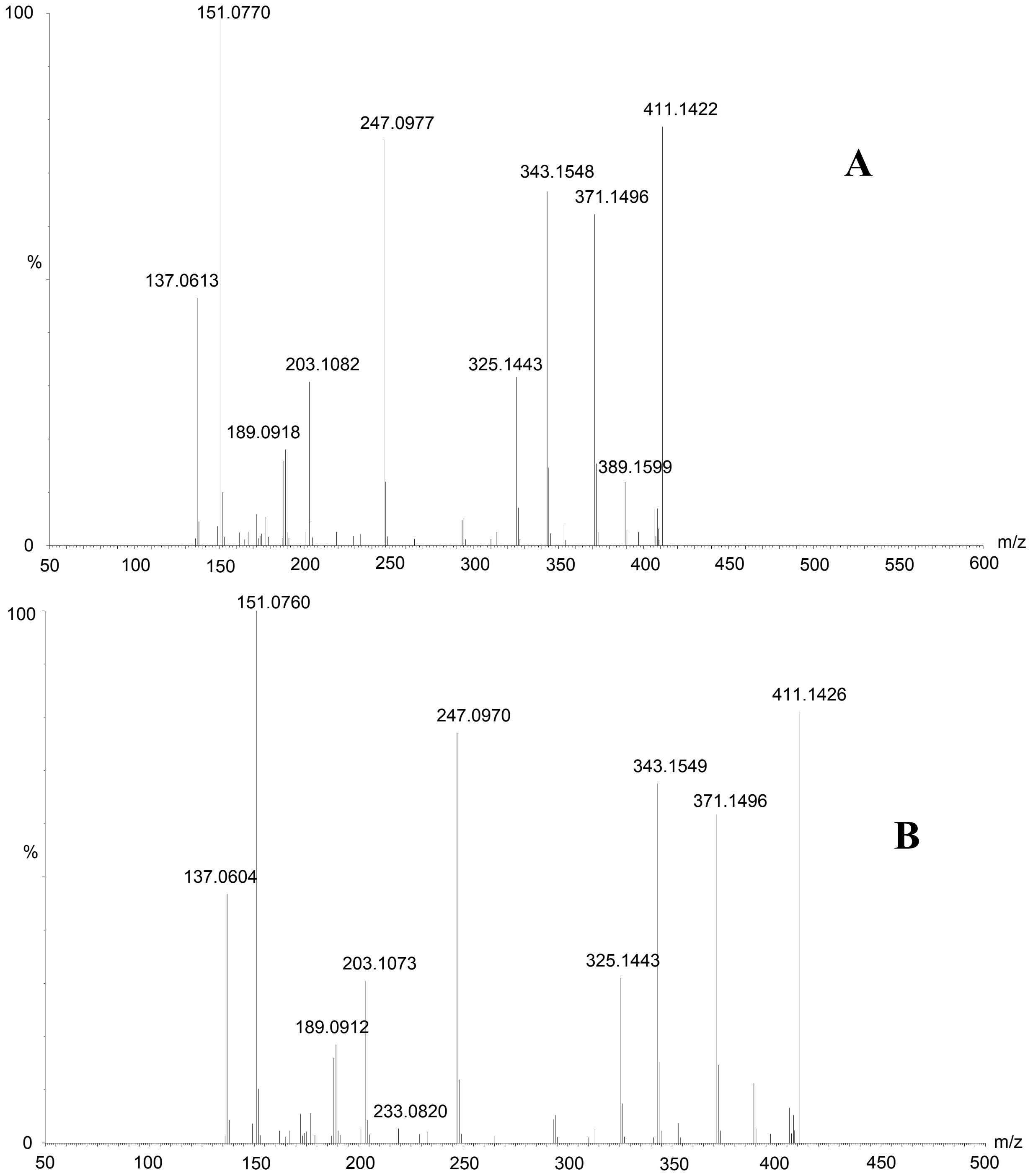

Figure S12. MS/MS spectrum of trachelogenin (**12**) in the standard sample (**A**) and in the sample extracted from Caulis Trachelospermi (**B**), respectively.

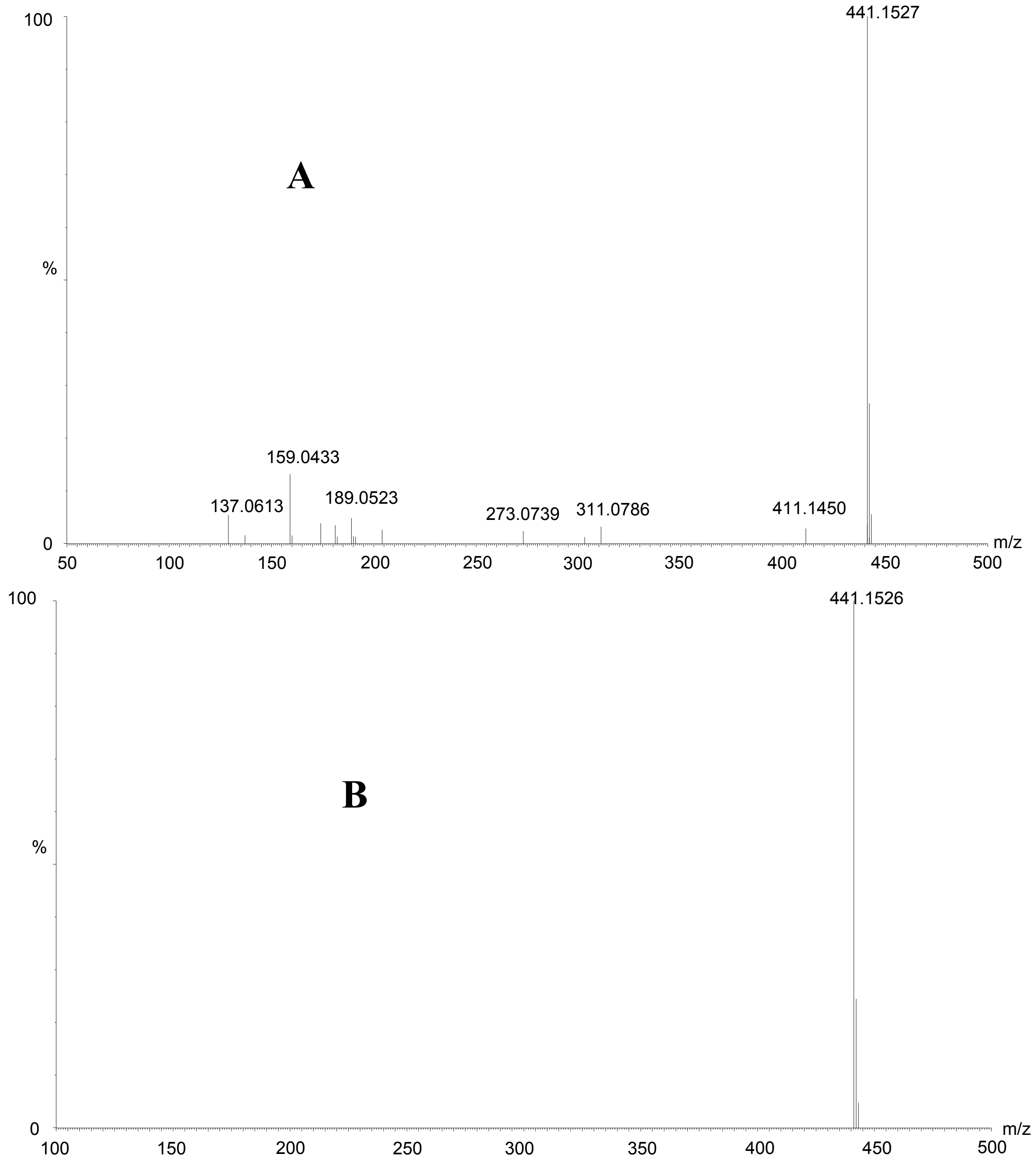

Figure S13. MS/MS spectrum of 5-methoxytrachelogenin (**13**) in the standard sample (**A**) and in the sample extracted from Caulis Trachelospermi (**B**), respectively.

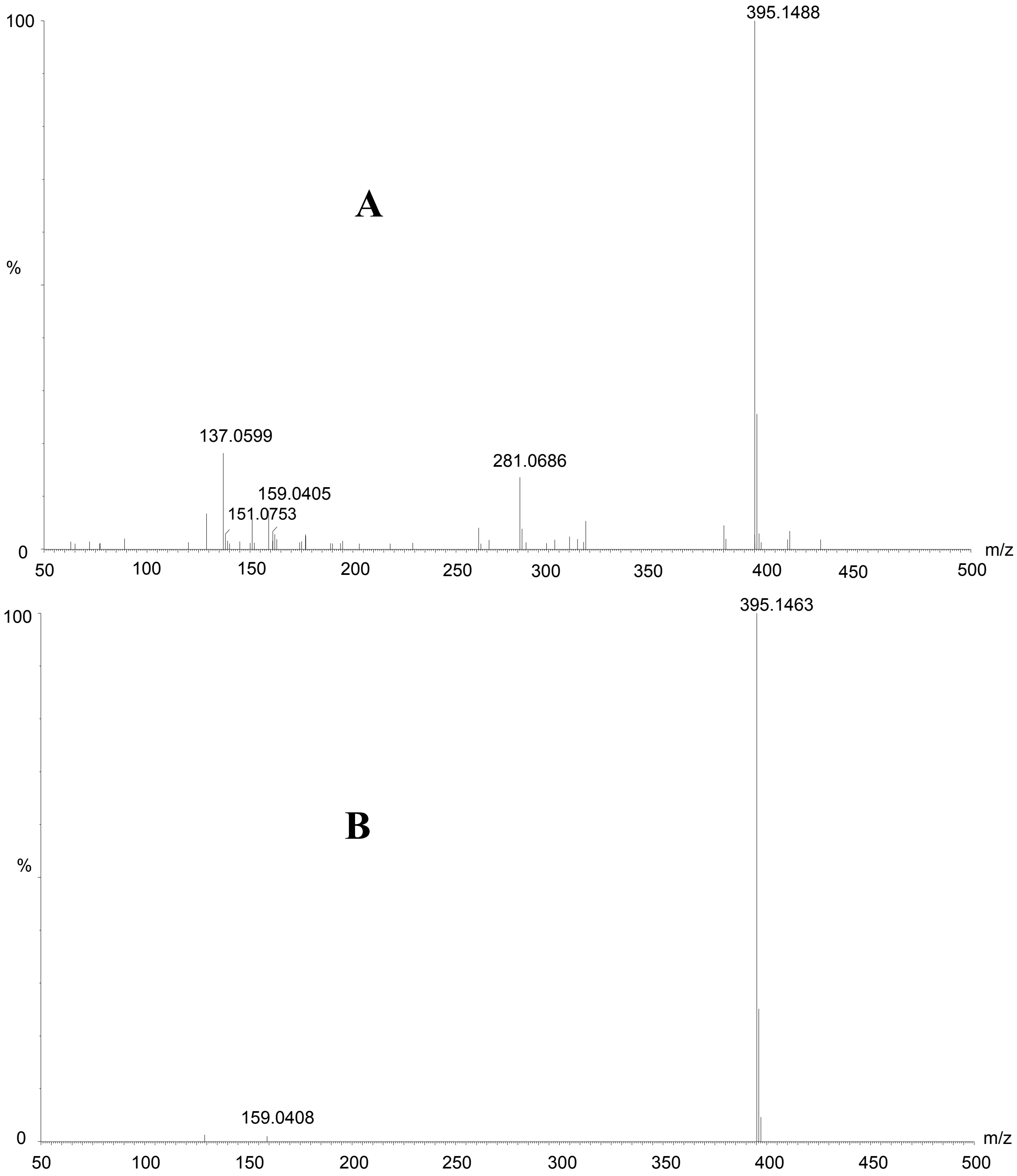

Figure S14. MS/MS spectrum of arctigenin (**14**) in the standard sample (**A**) and in the sample extracted from Caulis Trachelospermi (**B**), respectively.

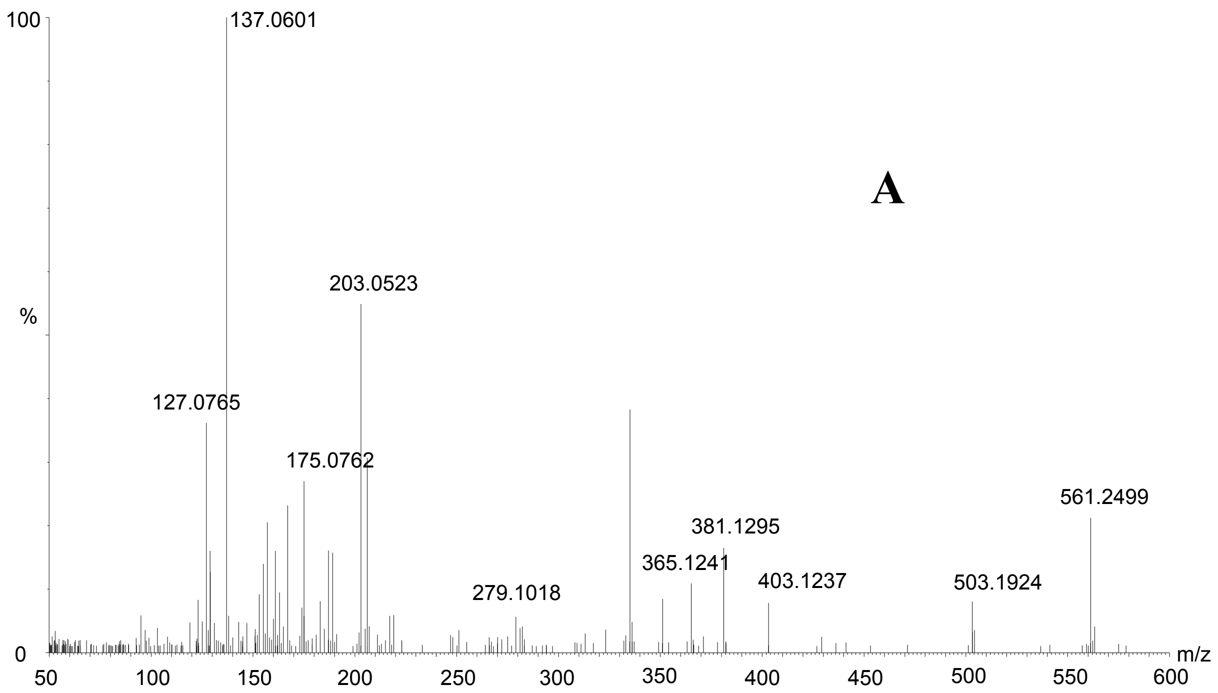

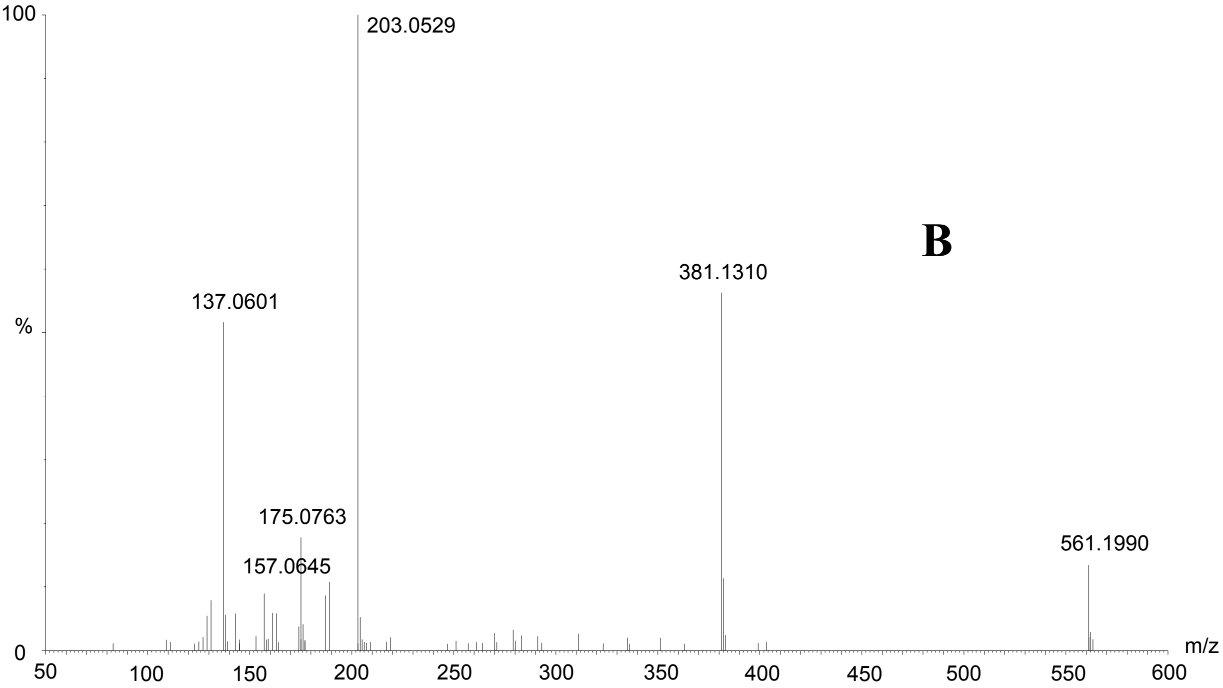

Figure S15. MS/MS spectrum of tanegoside A (**17**) in the standard sample (**A**) and in the sample extracted from Caulis Trachelospermi (**B**), respectively.

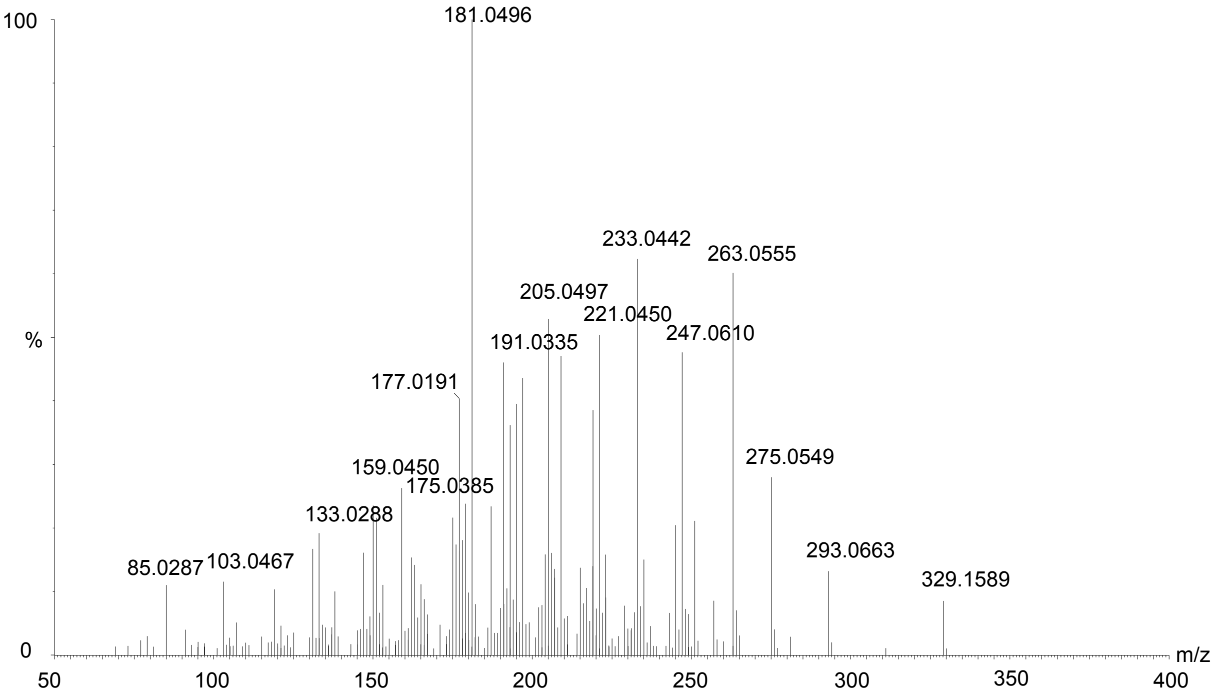

Figure S16. MS/MS spectrum of bergenin (**15**) in the sample extracted from Caulis Trachelospermi.

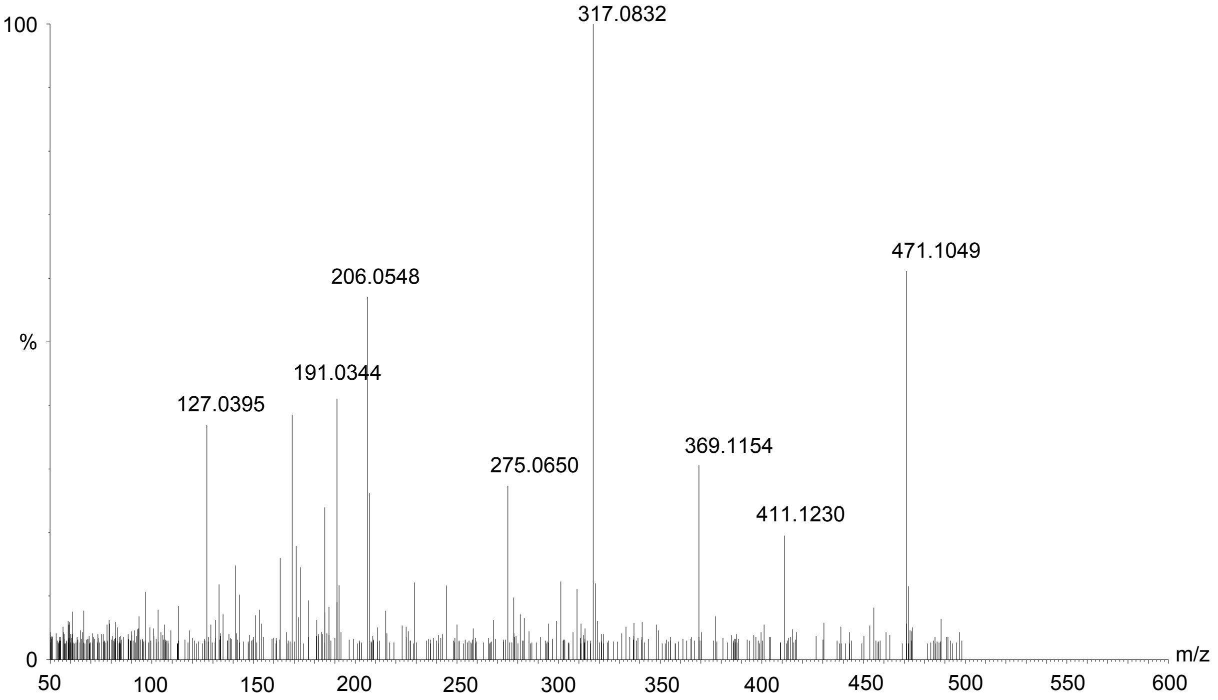

Figure S17. MS/MS spectrum of kelampayoside A (**16**) in the sample extracted from Caulis Trachelospermi.

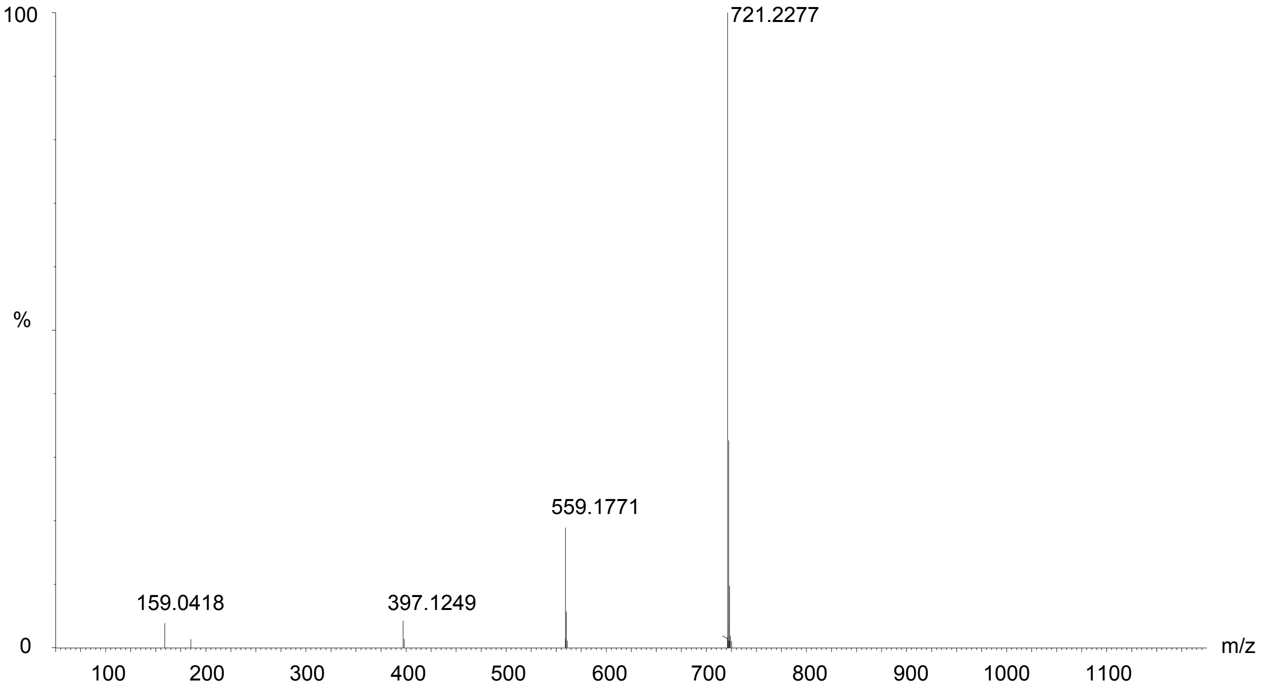

Figure S18. MS/MS spectrum of nortrachelogenin 4, 4'-di-*O*-*β*-D-glucoside (**18**) in the sample extracted from Caulis Trachelospermi.

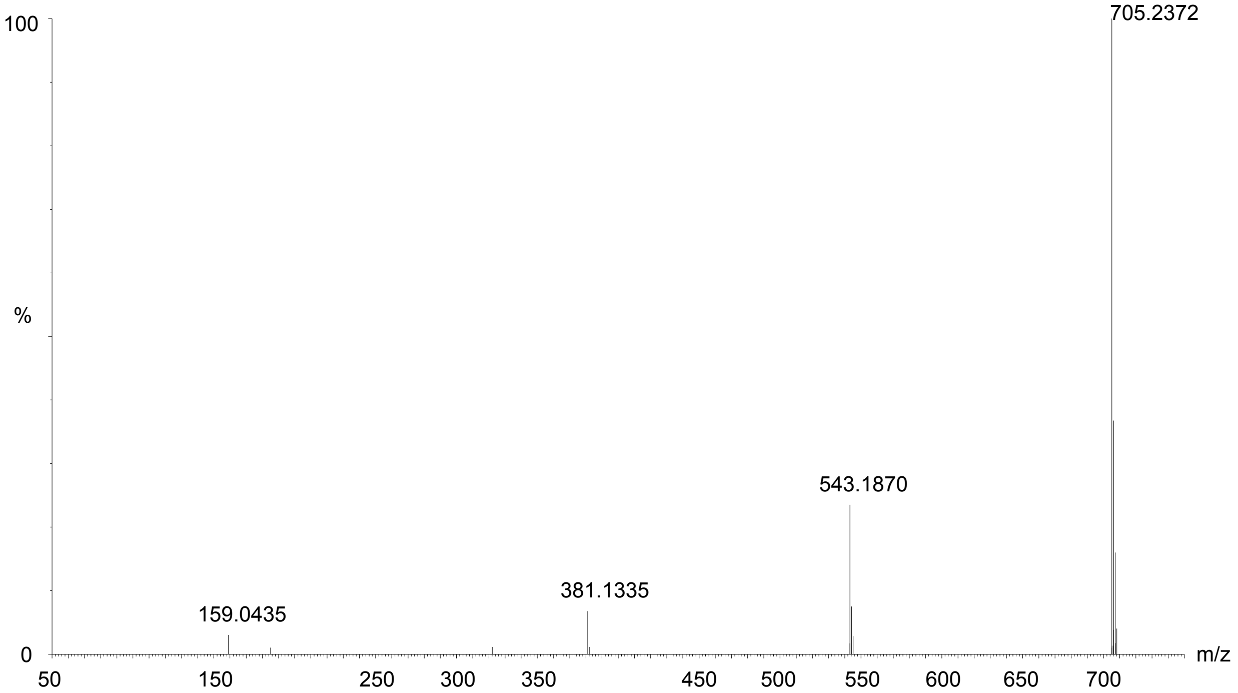

Figure S19. MS/MS spectrum of matairesinol 4, 4'-di-*O*-*β*-D-glucoside (**19**) in the sample extracted from Caulis Trachelospermi.

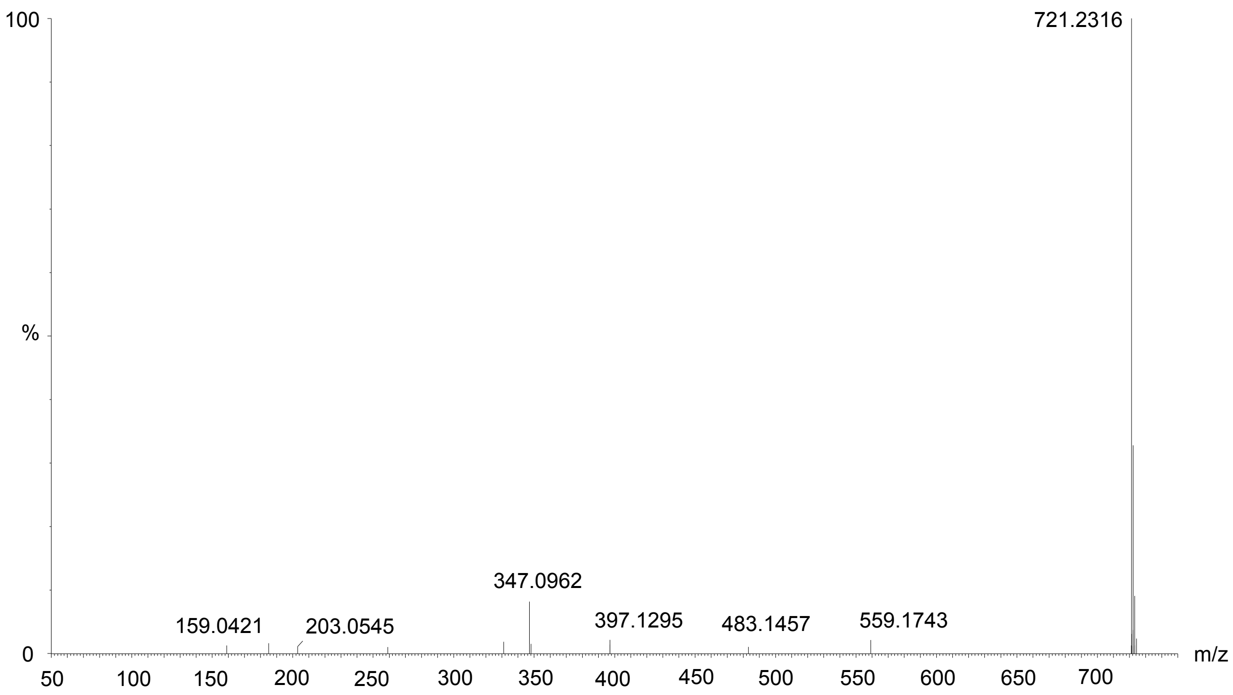

Figure S20. MS/MS spectrum of nortrachelogenin 4'-*O*-*β*-gentiobioside (**20**) in the sample extracted from Caulis Trachelospermi.

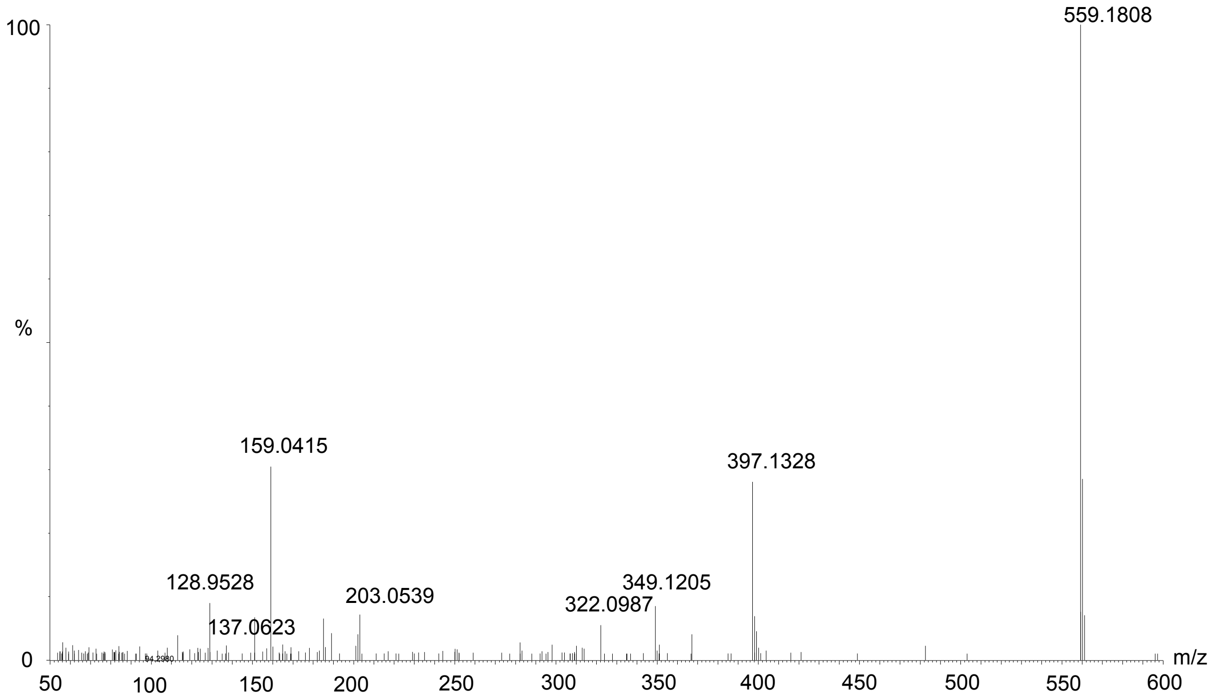

Figure S21. MS/MS spectrum of nortrachelogenin 4-*O*-*β*-D-glucoside (**21**) in the sample extracted from Caulis Trachelospermi.

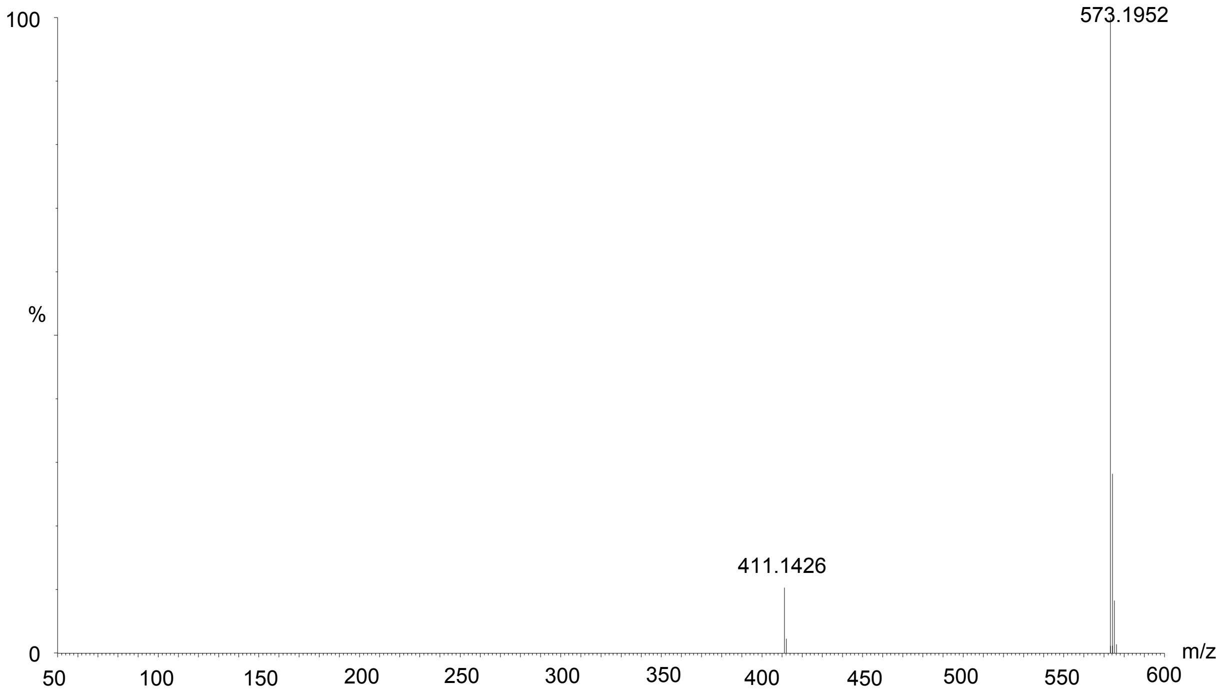

Figure S22. MS/MS spectrum of 4-demethyltraxillaside (**22**) in the sample extracted from Caulis Trachelospermi.

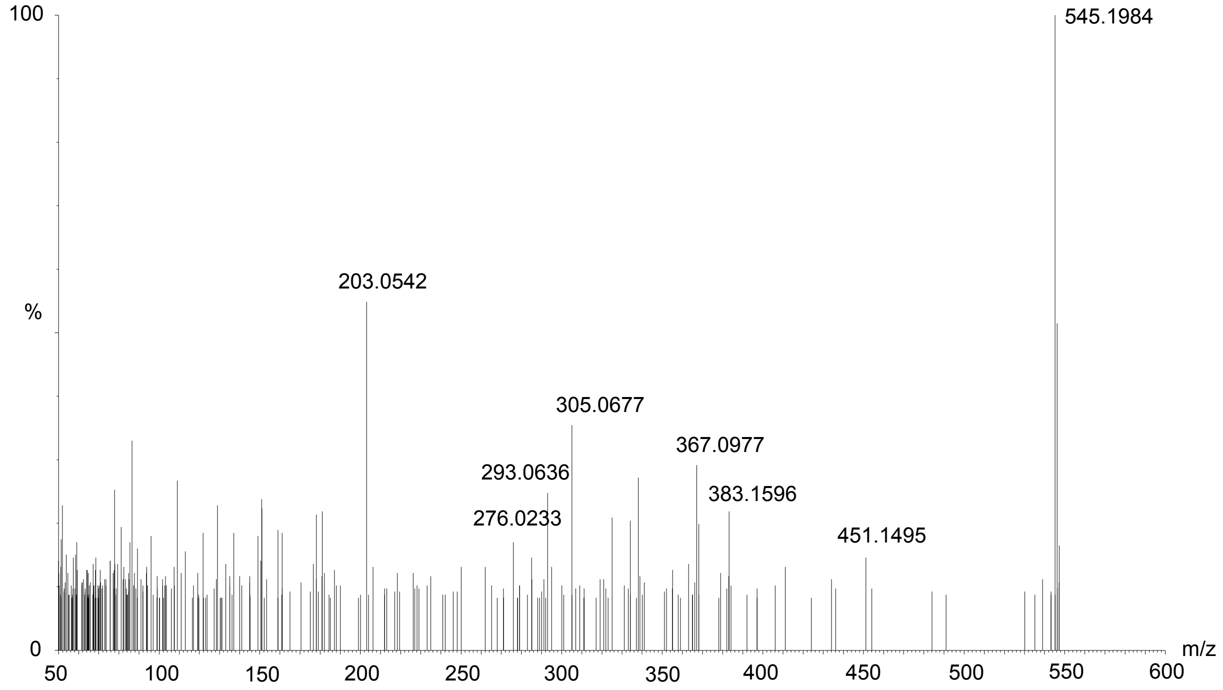

Figure S23. MS/MS spectrum of dihydrodehydrodiconiferyl alcohol-9-*O*-*β*-D-glucoside (**23**) in the sample extracted from Caulis Trachelospermi.

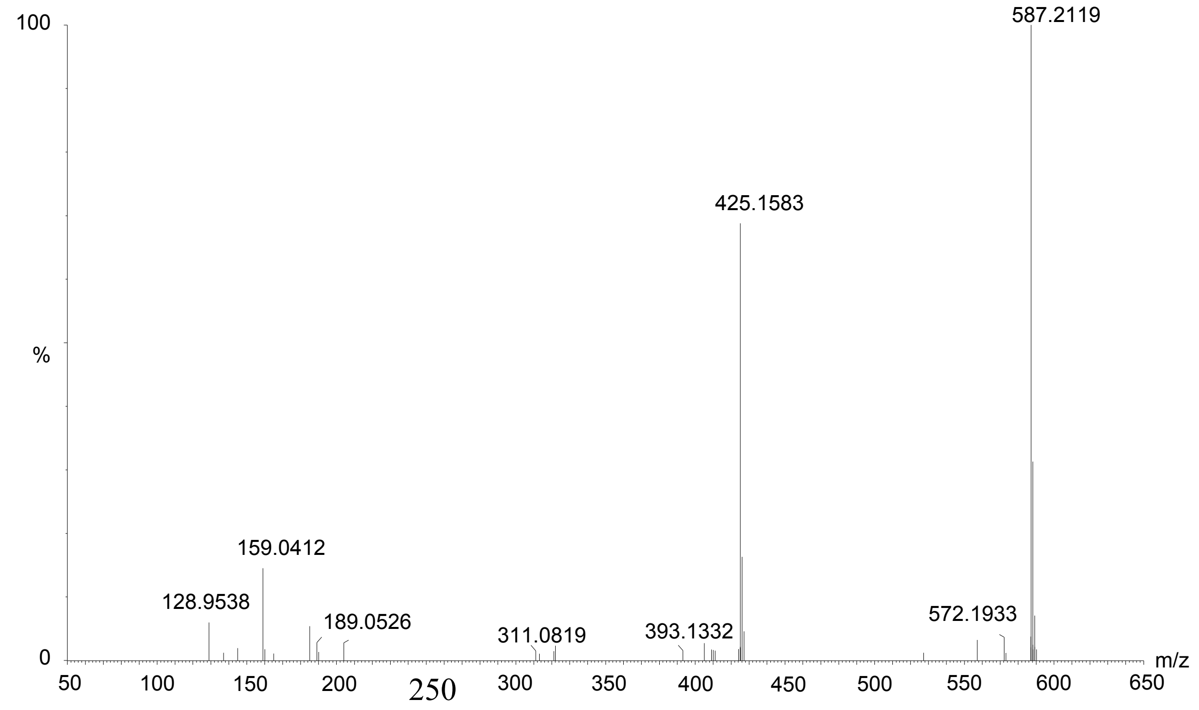

Figure S24. MS/MS spectrum of traxillageside (**24**) in the sample extracted from Caulis Trachelospermi.

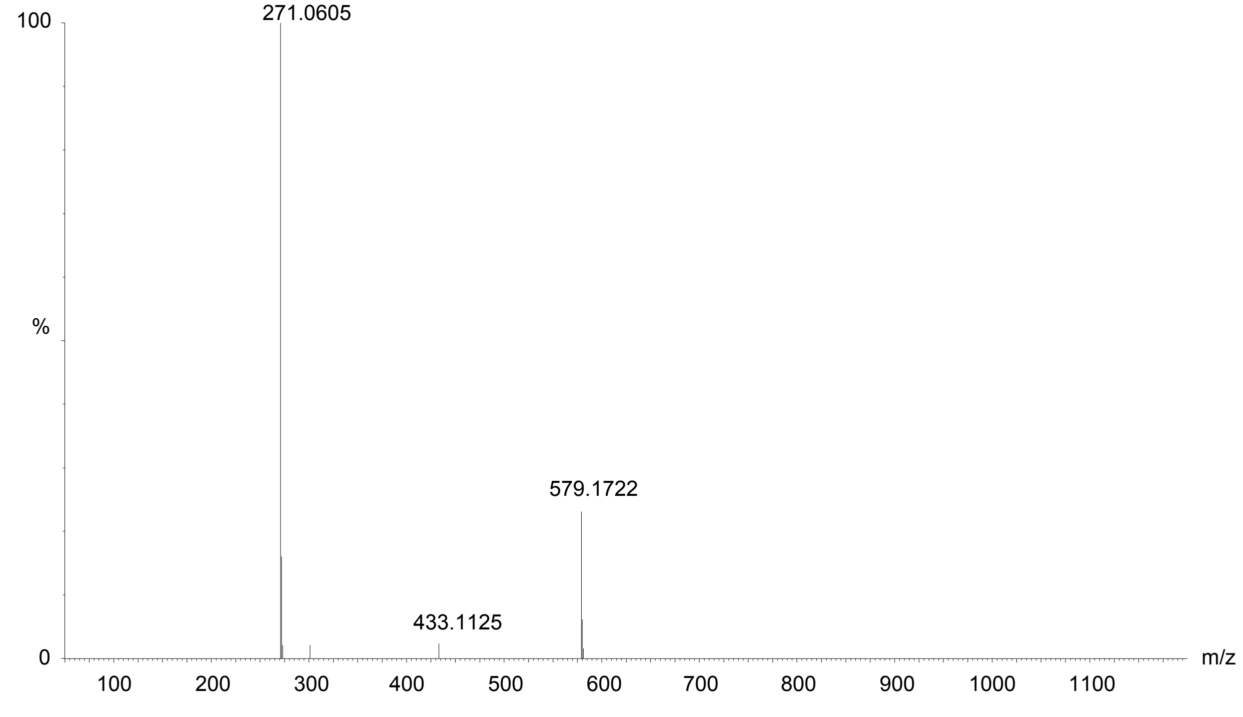

Figure S25. MS/MS spectrum of apigenin 7-*O*-*β*-neospheroside (**25**) in the sample extracted from Caulis Trachelospermi.
